# Transcriptome of Two Canine Prostate Cancer Cells Treated With Toceranib Phosphate Reveals Distinct Antitumor Profiles Associated With the PDGFR Pathway

**DOI:** 10.3389/fvets.2020.561212

**Published:** 2020-11-26

**Authors:** Priscila E. Kobayashi, Patrícia F. Lainetti, Antonio F. Leis-Filho, Flávia K. Delella, Marcio Carvalho, Sarah Santiloni Cury, Robson Francisco Carvalho, Carlos E. Fonseca-Alves, Renée Laufer-Amorim

**Affiliations:** ^1^Department of Veterinary Clinic, School of Veterinary Medicine and Animal Science, São Paulo State University—UNESP, Botucatu, Brazil; ^2^Department of Veterinary Surgery and Anesthesiology, School of Veterinary Medicine and Animal Science, São Paulo State University—UNESP, Botucatu, Brazil; ^3^Department of Morphology, Institute of Biosciences, São Paulo State University—UNESP, Botucatu, Brazil; ^4^Institute of Health Sciences, Paulista University—UNIP, Bauru, Brazil

**Keywords:** dog, prostate, microarray, animal model, antitumor response

## Abstract

Canine prostate cancer (PC) presents a poor antitumor response, usually late diagnosis and prognosis. Toceranib phosphate (TP) is a nonspecific inhibitor of receptor tyrosine kinases (RTKs), including vascular endothelial growth factor receptor (VEGFR), platelet-derived growth factor receptor (PDGFR), and c-KIT. This study aimed to evaluate VEGFR2, PDGFR-β, and c-KIT protein expression in two established canine PC cell lines (PC1 and PC2) and the transcriptome profile of the cells after treatment with TP. Immunofluorescence (IF) analysis revealed VEGFR2 and PDGFR-β protein expression and the absence of c-KIT protein expression in both cell lines. After TP treatment, only the viability of PC1 cells decreased in a dose-dependent manner. Transcriptome and enrichment analyses of treated PC1 cells revealed 181 upregulated genes, which were related to decreased angiogenesis and cell proliferation. In addition, we found upregulated *PDGFR-A, PDGFR-*β, and *PDGF-D* expression in PC1 cells, and the upregulation of *PDGFR-*β was also observed in treated PC1 cells by qPCR. PC2 cells had fewer protein-protein interactions (PPIs), with 18 upregulated and 22 downregulated genes; the upregulated genes were involved in the regulation of parallel pathways and mechanisms related to proliferation, which could be associated with the resistance observed after treatment. The canine PC1 cell line but not the PC2 cell line showed decreased viability after treatment with TP, although both cell lines expressed PDGFR and VEGFR receptors. Further studies could explain the mechanism of resistance in PC2 cells and provide a basis for personalized treatment for dogs with PC.

## Introduction

Although the prevalence of prostate cancer (PC) in dogs is relatively low, canines are the only domestic species other than humans known to spontaneously develop PC (1, 2). Canine prostate carcinoma is a biologically aggressive neoplasm that exhibits a poor prognosis related to its late diagnosis, high metastatic rate (80% at death), and limited effective treatments (1, 3, 4). Some reported metastatic sites include lung, bone, lymph node, liver, spleen, and colon (4–8).

In contrast to PC in humans, PC in dogs is not androgen dependent, so androgen deprivation therapy is not effective (9). Due to some important differences between PC in men and dogs, many treatment modalities used successfully in human medicine cannot be applied in dogs (9). Therefore, there is a need for new therapies for canine PC, including targeted therapies, which are drugs that target specific proteins in neoplastic cells or tumor-associated antigens and exert less damage to normal cells (10).

Toceranib phosphate (TP), the veterinary counterpart to sunitinib (SU11248), works by preventing receptor tyrosine kinase (RTK) phosphorylation and consequent downstream signaling molecules (11–13). TP is a nonspecific RTK inhibitor with targets including VEGFR (vascular endothelial growth factor receptor), PDGFR (platelet-derived growth factor receptor), and c-KIT; this drug was approved for the treatment of dogs with cutaneous mast cell tumors and was originally developed as an antiangiogenic agent (14–16). Additionally, some phase I clinical trials in dogs suggest that TP treatment exhibits clinical antitumor activity against different cancers, including gastrointestinal stromal tumors, lymphomas, multiple myelomas, metastatic soft tissue sarcomas, and several carcinomas, such as metastatic mammary, head and neck, thyroid, and prostatic carcinomas (11, 17–19).

Although some studies have demonstrated the clinical anticancer effect of TP in dogs, the mechanisms of action have not been elucidated. We used global gene expression analysis in two canine PC cell lines to evaluate the genes involved in the treatment response to TP. To investigate the molecular mechanism underlying TP cytotoxicity in canine PC cell lines, we evaluated cell viability and gene expression alterations in response to TP.

## Materials and Methods

### Ethical Approval

This study was approved by the Ethics Committee on Animal Use (CEUA) of the School of Veterinary Medicine and Animal Science of the São Paulo State University, Botucatu (Protocol: 0004/2017). Written informed consent was obtained from the owners for the participation of their animals in this study.

### Canine Primary PC Cell Culture

Two characterized canine primary prostate cancer cell cultures (PC1 and PC2) from two different canine PCs collected during necropsy, were cultured as previously described (20). PC1 cell was stablished from prostate carcinoma of a 10-year- old, intact, mixed breed dog and PC2 from an 11-year-old, intact poodle dog. PC1 and PC2 cells were cultured at 37°C in 5% CO_2_ in complete medium comprising DMEM/F12 (Lonza Inc., Allendale, NJ, USA), 10% inactivated fetal bovine serum (FBS, HYCLONE, Waltham, MA, USA), 100 U/mL penicillin G and 100 mg/mL streptomycin (Sigma, Portland, OR, USA).

### Assessment of Protein Expression by Immunofluorescence (IF)

To evaluate the effects of TK receptors in these cell lines, we performed IF for VEGFR2, PDGFR-β, and c-KIT. PC1 and PC2 cells were seeded at 5 × 10^4^ cells/well into 12-well chamber slides (SPL Life Sciences) and allowed to grow on coverslips at 37°C and 5% CO_2_ until they reached ~70% confluence. Cells were then washed using Dulbecco's phosphate-buffered saline (DPBS) at 4°C and fixed in methanol for 30 min at 27°C. Samples were permeabilized with 0.1% Triton X-100 in PBS for 10 min at 27°C and incubated with a commercial reagent (Protein Block Serum Free, Dako®) for 30 min to block nonspecific antibody binding. The cells were incubated for 18 h at 4°C in a humidified atmosphere with primary antibodies against VEGFR2 (Clone SC-6251, Santa Cruz Biotechnology), PDGFR-β (Clone 3162, Cell Signaling Technology®), and c-KIT (Clone A4502, Dako®), all of which were diluted 1:100. Next, the cells were incubated with secondary antibodies at 1:10,000 dilutions and conjugated to the following fluorophores: Alexa Fluor 594 (Clone Poly4053, BioLegend) for VEGFR and Alexa Fluor® 488 (Clone A11034, ThermoFisher Scientific) for PDGFR-β and c-KIT (CD117). The samples were then labeled with DAPI (4,6-diamidino-2-phenylindole dihydrochloride) (D9542, Dako®) at a 1:10,000 dilution to stain the nuclei and examined and imaged under a TCS SP5 confocal microscope (Leica Biosystems, Wetzlar, Alemanha).

PC cell lines were considered to have positive or negative expression according to the immunofluorescent results.

### MTT Assay and IC50 Detection

PC1 and PC2 cell lines were seeded into 96-well-plates at a concentration of 1 × 10^4^ cells/well in 0.1 mL of complete medium and allowed to grow for 24 h in a 5% CO_2_ incubator. The medium was removed and replaced with serum-free medium containing different concentrations of TP (Sigma-Aldrich; 3, 6, 9, and 12 μM; 12 wells per concentration) dissolved in dimethyl sulfoxide (DMSO—Hybri-Max™; Sigma). Two control groups—no treatment and 0.4% DMSO (vehicle control), were also established to confirm that the vehicle had no influence on cell viability. The wells were incubated for 24, 48, and 72 h after the addition of the treatments.

An MTT stock solution was prepared with 0.013 g of MTT (Invitrogen™, M6494) dissolved in PBS (2.5 mL); 1 mL of the stock solution was diluted 10-fold in serum-free medium to establish a final concentration of 0.5 mg/mL. After the addition of MTT, the plates were incubated at 37°C for 4 h. Next, the medium was removed from all the plates, and the precipitated MTT salts were dissolved with 200 μL of DMSO per well for 15 min. Absorbance values at 595 nm were recorded with a multiwell plate reader.

Cell viability was calculated as a percentage using the following formula: (A_treatment_ – A_blank_)/(A_DMSO_ – A_blank_) × 100% (21), with *A* = absorbance, DMSO = vehicle control, and blank = no cells. The IC50 values were calculated using Graph Pad Prism 8.0 from a log ([drug]) vs. normalized response curve fit.

### Quantitative PCR

After establishing the IC_50_ value of TP for each cell line, we treated PC1 and PC2 cells with the IC_50_ (treated cells) for 24 h and extracted RNA for RT-qPCR and transcriptome analysis.

This assay was performed in duplicate, and, as a control, an equivalent volume of DMSO alone was added to cells (nontreated cells). Isolation and purification of total RNA were performed with a commercial kit according to the manufacturer's instructions (RNeasy mini kit, Qiagen, Hilden, Germany). The RNA concentration and purity were evaluated by spectrophotometry (NanoDrop™, ND-8000, Thermo Scientific, Waltham, MA, USA) whereas the RNA integrity was assessed by the Bioanalyzer 2100 and the Agilent RNA 6000 Nano Series kit according to the manufacturer's instructions (Agilent Technologies, Santa Clara, CA, USA).

cDNA synthesis was carried out using 1 μg of total RNA treated with DNAse I (Life Technologies, Rockville, MD, USA), 200 U of *SuperScript* III Reverse Transcriptase enzyme (Life Technologies), 4 μL of SuperScript First-Strand Buffer 5X, 1 μL each of 10 mM dNTP (Life Technologies), 1 μL of Oligo-(dT)_18_ (500 ng/μL) (Life Technologies), 1 μL of random hexamers (100 ng/μL) (Life Technologies), and 1 μL of 0.1 M DTT (Life Technologies). Reverse transcription was performed at 50°C for 60 minutes, and the reactions were inactivated at 70°C for 15 min. qPCR amplification for *VEGFR2, PDGFR-*β, and *KIT* as well as for reference genes (*GAPDH, HPRT, RPS19, RPS5*, and *RPL8)* was performed using QuantStudio 12k Flex Thermal Cycler equipment (Applied Biosystems; Foster City, CA, USA). The reactions were performed in duplicate in 384-well-plates using Power SYBR Green PCR Master Mix (Applied Biosystems; Foster City, CA, USA), 1 μL of cDNA, and 0.3 μM of each primer. Relative gene quantification was calculated by the 2^−ΔΔ*CT*^ method (22).

### Microarray

We generated a global gene expression profile (microarray) using GeneChip® Canine Gene 1.0 ST Arrays (Affymetrix, CA, EUA). cDNA labeling, hybridization, and detection were performed according to the manufacturer's instructions. Then, the chips were scanned in a Scanner 3000 7G series (Affymetrix, Santa Clara, CA, EUA). Affymetrix CEL files were downloaded and processed with Applied Biosystem™ Transcriptome Analysis Console (TAC, Affymetrix) software. The criteria for selecting differentially expressed genes (DEGs) were a 2.0-fold change cutoff and a *P* < 0.05. Hierarchical clustering heatmaps and Venn diagrams were generated using TAC software.

### Gene Ontology (GO)

The DEGs between the groups were subjected to a GO enrichment analysis using Enrichr (https://amp.pharm.mssm.edu/Enrichr/). REVIGO (http://revigo.irb.hr/) was used to organize and visualize the enriched GO terms obtained from Enrichr. GO analysis was focused on two major categories: biological process and molecular function.

### Protein-Protein Interaction (PPI) Networks

The upregulated and downregulated DEGs were independently submitted to the online Search Tool for the Retrieval of Interacting Genes—STRING (https://string-db.org/) to generate PPI networks. We considered only STRING interactions with high confidence (0.700), and active interactions were defined as databases, coexpression, neighborhood, and cooccurrence. To simplify the network, we hid the disconnected nodes.

### Transcriptomic Analysis of Primary Canine Prostate Tumors

To evaluate the expression profile of PC1 and PC2 in primary tumors we downloaded the RNAseq data from GSE122916 study available at GEO (Gene Expression Omnibus) database (23). We then performed differential expression analysis using the NetworkAnalyst 3.0 software (24–26). Nine malignant were compared with nine non-malignant prostate tissues (biopsy) and two malignant were independently compared to five non-malignant prostate tissues (fine-needle-aspiration). Differentially expressed genes of prostate cancer were identified using EdgeR (27). The HTCounts were normalized using a trimmed mean of M-values (TMM). Genes were filtered out when presenting low abundance (less than four counts) and stable expression across conditions. We selected the genes with |logFC| > 1 and adjusted *p* < 0.05 regulated in the same direction in both biopsy and fine-needle-aspiration tumor samples.

We used the Set Comparison Appyter v0.0.6 online tool (https://appyters.maayanlab.cloud/#/CompareSets) to determine whether the overlaps between PC1/PC2 with primary tumors are significant.

### Statistical Analysis

Comparisons among the different doses in the treatment groups were made using the Tukey–Kramer test, and statistical significance was set at *p* < 0.05. *S*tatistical analysis was performed, and graphs were generated using Graph Pad Prism 8.0 and Microsoft® Excel 2007.

## Results

### Measurement of Protein Expression by IF

**Figure 1:**
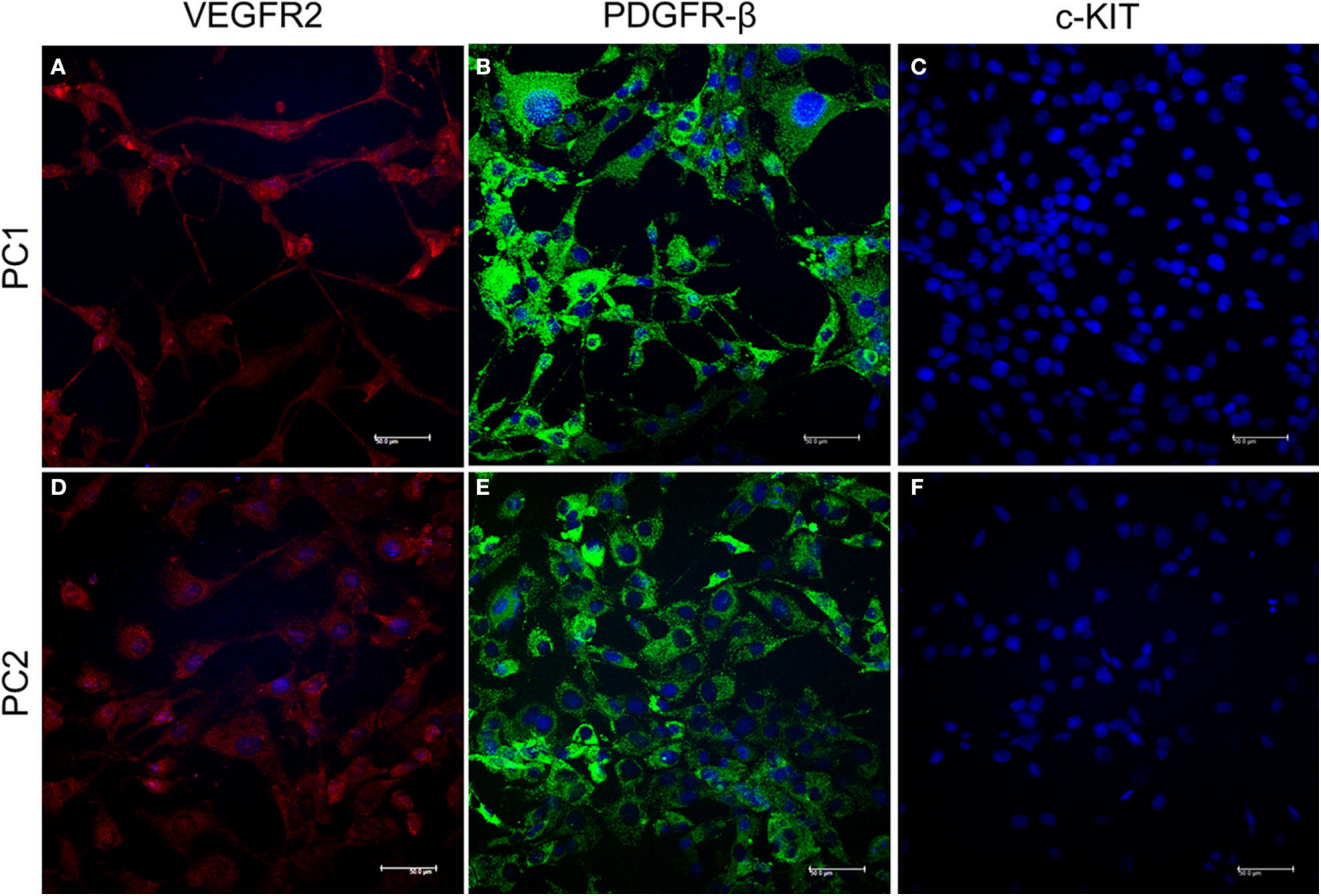
Detection of VEGFR2, PDGFR-β, and c-KIT protein expression by IF. Cytoplasmic expression of VEGFR2 is indicated by red fluorescence **(A,B)** and PDGFR-β by green fluorescence **(C,D)** in PC1 and PC2 cells, respectively. No c-KIT protein expression **(E,F)** was observed in either cell line. The nuclei were counterstained with DAPI (blue).

PC1 and PC2 cells showed VEGFR2 and PDGFR-β cytoplasmic expression. However, c-KIT protein was absent in both cell lines (Figure 1).

### MTT Assay and IC50 Detection

**Figure 2:**
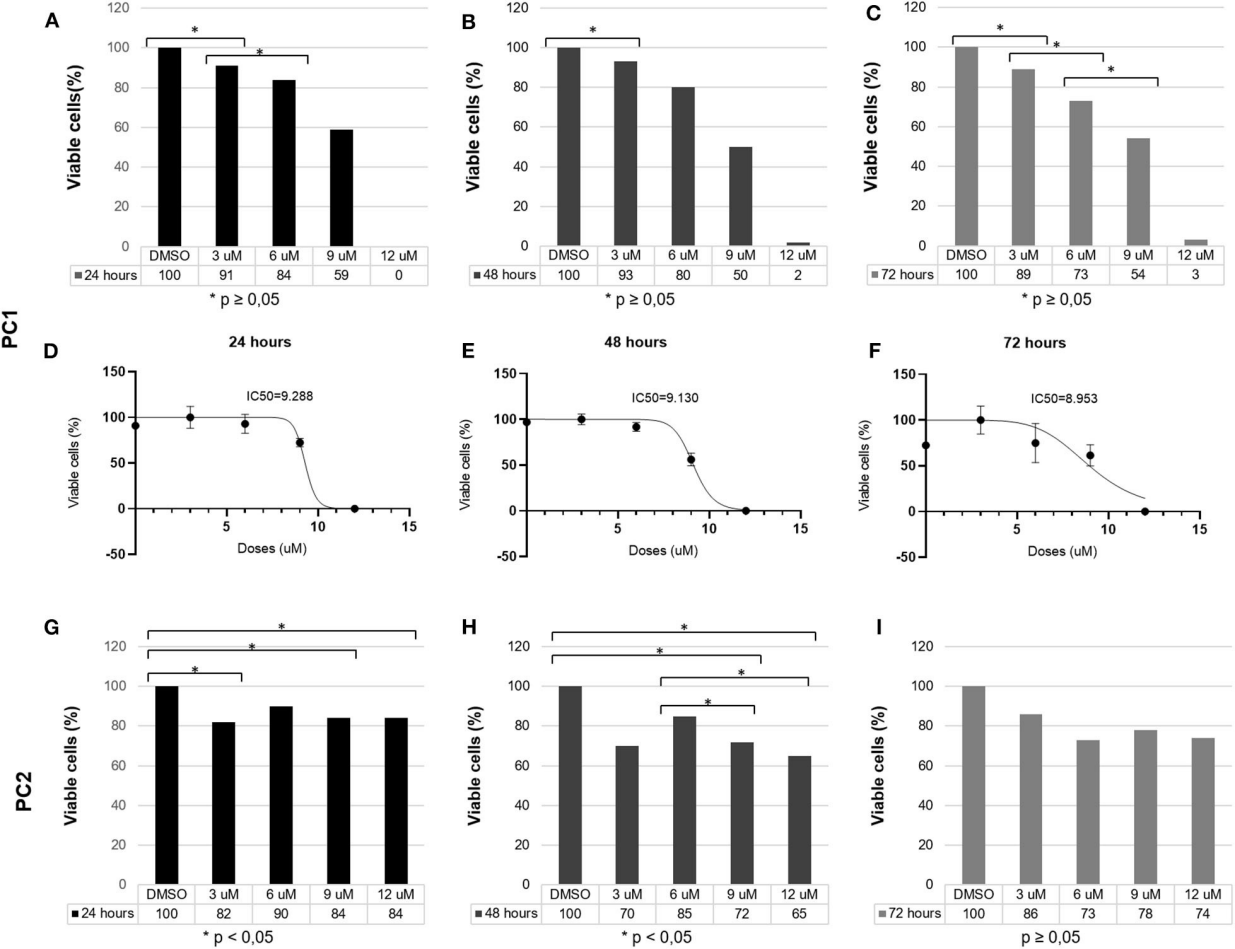
PC1 and PC2 cells were cultured with various concentrations of TP (3, 6, 9, and 12 μM), and cell viability was assessed using an MTT assay. PC1 cells were treated with TP for **(A)** 24, **(B)** 48, and **(C)** 72 h. The IC_50_ values of TP after **(D)** 24, **(E)** 48, and **(F)** 72 h of incubation in PC1 cells. PC2 cells were treated with TP for **(G)** 24, **(H)** 48, and **(I)** 72 h. A value of *p* < 0.05 was considered statistically significant. The symbol * corresponds of *p* value.

TP reduced PC1 cell viability in a dose-dependent manner. After 24 h, 3, 6, 9, and 12 μM TP reduced the viability of PC1 cells to 91, 84, 59, and 0%, respectively; after 48 h, the cell viability was reduced to 93, 80, 50, and 2%; and after 72 h, the cell viability was reduced to 89, 73, 54, and 3% (Figure 2). Following treatment with TP for 24 (Figure 2A), 48 (Figure 2B), and 72 h (Figure 2C), a significant (*p* < 0.05) decrease in the number of viable cells was observed in PC1 cells compared to that of the control cells (DMSO). The IC_50_ values of TP in PC1 cells were 9.28, 9.13, and 8.95 μM at 24 (Figure 2D), 48 (Figure 2E), and 72 (Figure 2F) h, respectively.

**Figure 3:**
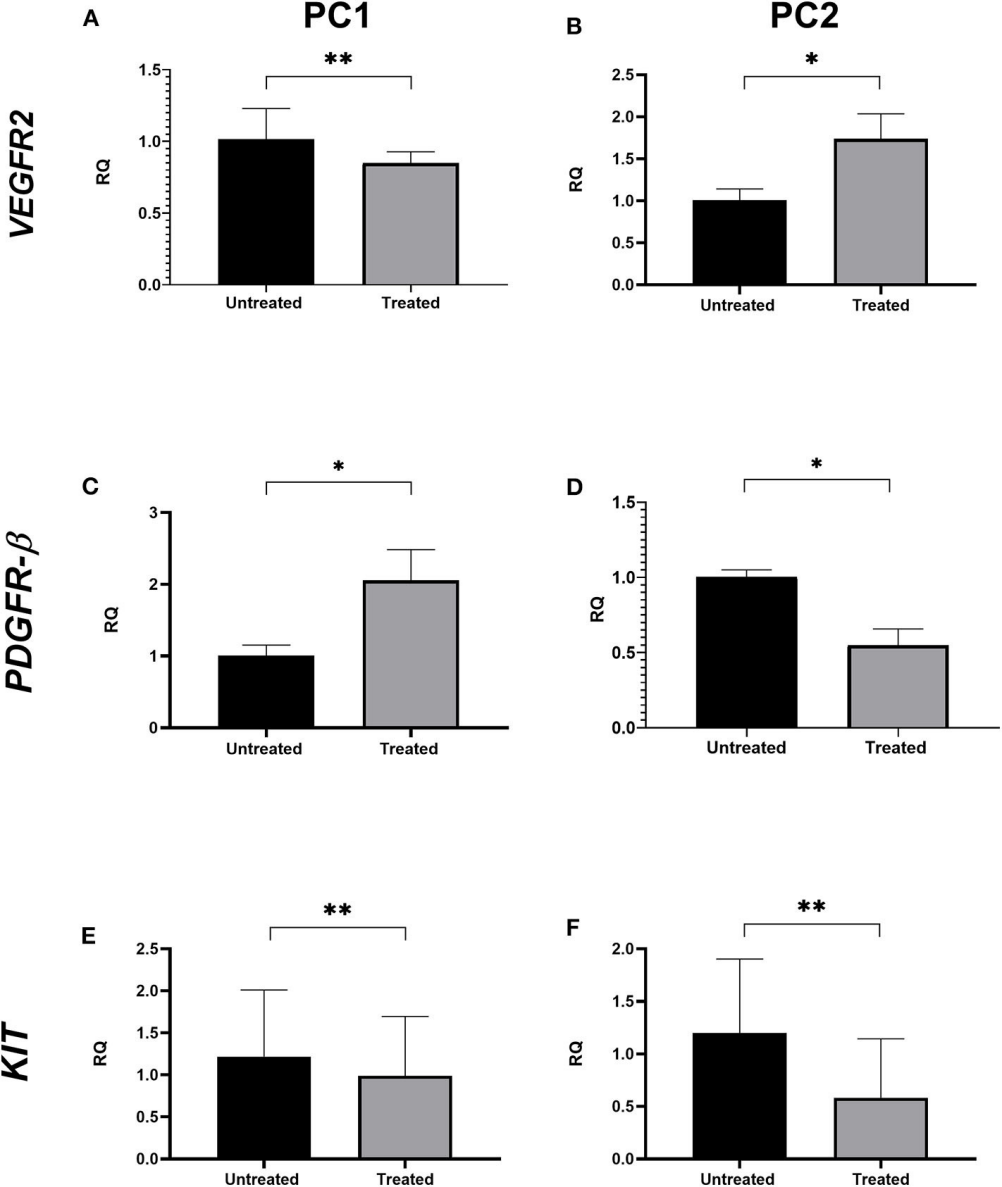
The effect of TP on *VEGFR2, PDGFR-*β, and *KIT* transcript levels in PC1 and PC2 cells. Relative quantification levels of *VEGFR2* **(A,B)**, *PDGFR-*β **(C,D)** and *KIT* **(E,F)** were measured by RT-qPCR in PC1 and PC2 cells treated with their respective IC50 value of TP or DMSO. *p < 0.05, **p ≥ 0.05.

On the other hand, TP did not reduce PC2 cell viability in a dose-dependent manner. After 24 h (Figure 2G), 3, 6, 9, and 12 μM TP reduced the viability of PC2 cells to 82, 90, 84, and 84%, respectively, compared to that of the control cells; after 48 h (Figure 2H), the cell viability was reduced to 70, 85, 72, and 65%; and after 72 h (Figure 2I), the cell viability was reduced to 86, 73, 78, and 74%. Thus, we considered the PC2 cell line to be more resistant to TP than the PC1 cell line. Lower toceranib phosphate doses concentrations (125, 250, 500nM, 1, 1.5 μM were tested in both cells (PC1 and PC2) with no cell viability alterations (data not shown).

### qPCR

*VEGFR2* transcript levels were statistically lower in treated PC1 cells (mean = 1.017) than in untreated cells (mean = 0.849; *p* < 0.05; Figure 3A); however, no significant differences were observed in *VEGFR2* gene expression between the untreated and treated PC2 cells (Figure 3B). There was a significant upregulation of *PDGFR-*β expression in treated PC1 cells (mean = 2.056) comparing to untreated PC1 cells (mean = 1.007) (Figure 3C). We observed downregulation of *PDGFR-*β expression in treated PC2 cells (mean = 0.549) compared with untreated PC2 cells (mean = 1.001; Figure 3D). However, treatment with TP for 24 h did not alter transcript *KIT* levels between untreated and treated cells for either PC1 or PC2 cells (Figures 3E,F, respectively).

### Transcriptome Analysis

A total of 390 DEGs (*p* < 0.05) were observed between untreated and TP-treated PC1 cells (233 upregulated genes and 157 downregulated genes in treated cells). However, 122 DEGs were not annotated with gene names or gene symbols in TAC software, and 5 were microRNAs.

**Figure 4:**
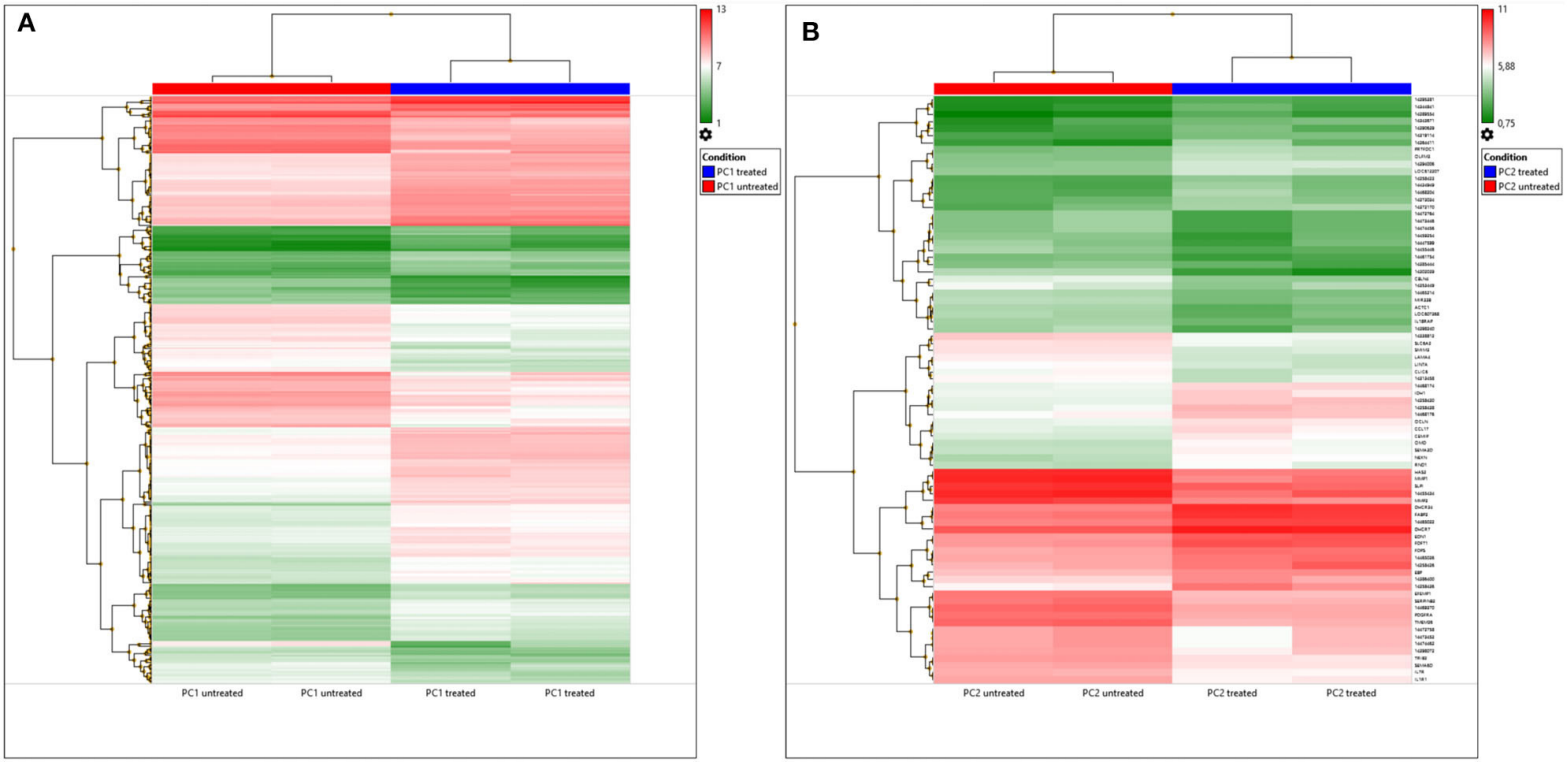
DEGs in untreated and TP-treated PC1 and PC2 cells based on microarray analysis. **(A)** Cluster heatmap: red represents upregulation whereas green indicates downregulation of gene expression relative to that in untreated cells. Each row represents a gene, and each column represents a sample. Each sample was analyzed in duplicate. **(B)** Venn diagram: overlapping sections show common genes deregulated by TP in PC1 (yellow) and PC2 (blue) cells compared to the respective untreated cells.

A total of 82 DEGs were observed (*p* < 0.05) in treated PC2 cells compared to control cells, including 42 upregulated genes and 40 downregulated genes. However, the DEG list showed that out of the 82, 42 genes had neither a gene name nor a symbol, and 1 gene was a microRNA. A heatmap of these DEGs is shown in Figure 4A, and DEGs were clustered, which can differentiate the treated and untreated PC1 and PC2 cells.

A Venn diagram (Figure 4B) was used to compare the DEGs between the PC1 (treated and untreated) and PC2 (treated and untreated) cells. From a total of 525 DEGs, only 17 common DEGs were screened out. Among these genes, *PDGFR-A* was altered in TP-treated PC1 and PC2 cells, but it was upregulated in PC1 cells and downregulated in PC2 cells. Treated PC1 cells also showed upregulation of *PDGFR-*β and *PDGF-D*. There are summarized lists of all the upregulated and downregulated genes in both treated PC1 cells (Supplementary Tables 1, 2, respectively) and treated PC2 cells (Supplementary Tables 3, 4, respectively).

### GO Analysis

**Figure 5:**
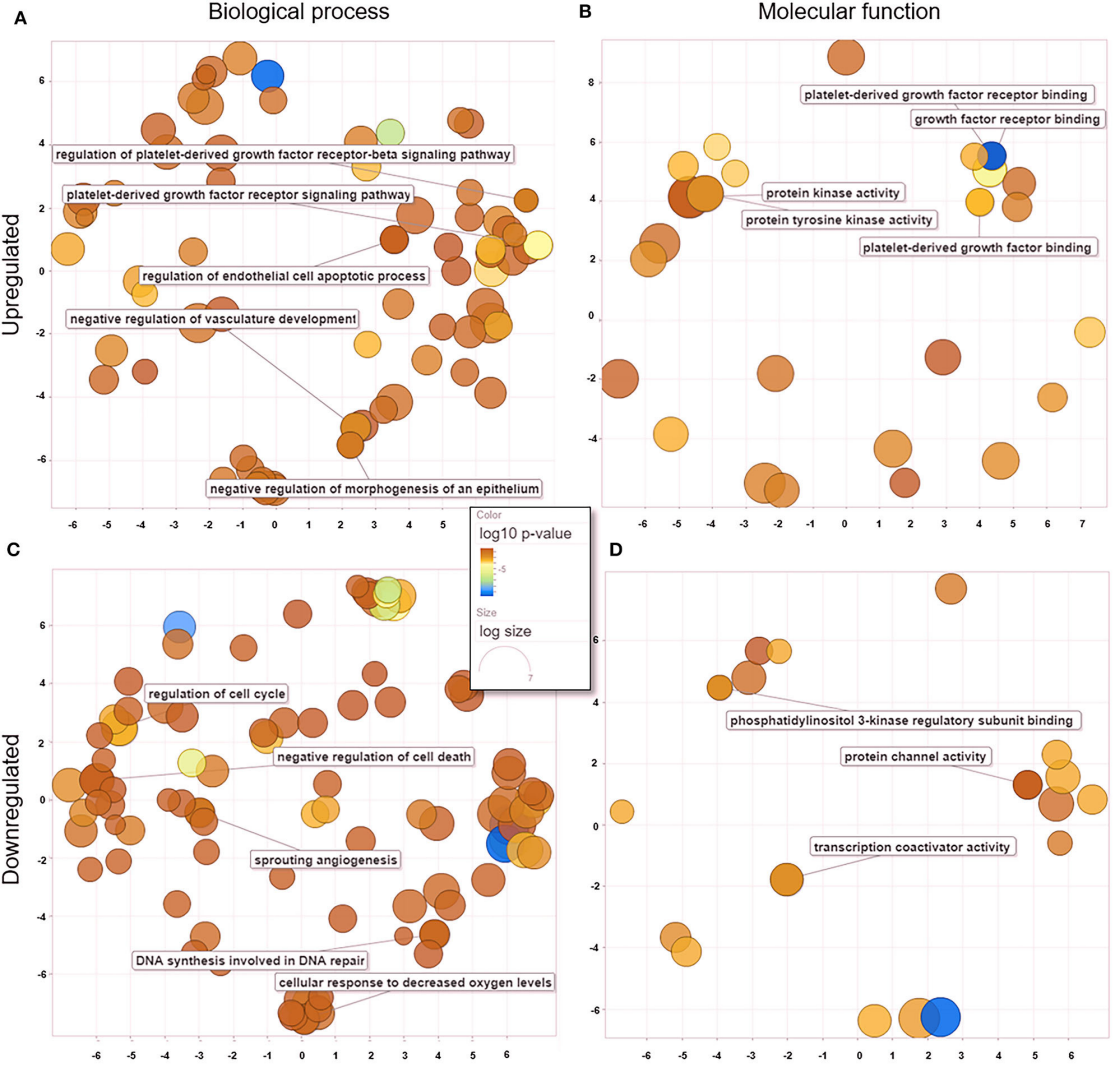
GO processes: common biological processes and molecular functions of both upregulated and downregulated DEGs in treated PC1 cells. **(A)** Biological processes of upregulated DEGs, **(B)** molecular functions of upregulated DEGs, **(C)** biological processes of downregulated DEGs, **(D)** molecular functions of downregulated DEGs. The scatter plot was produced with REVIGO software.

We analyzed GO data associated with DEGs in untreated and treated PC1 cells, and we observed 181 enriched genes that were significantly upregulated (*p* < 0.05) and 82 that were significantly downregulated (*p* < 0.05). Redundant GO terms with no statistically significant differences were removed using REVIGO. The analysis revealed that among the 167 biological processes (Figure 5A) associated with the upregulated genes in the TP-treated cells, regulation of the PDGFR and PDGFR-β signaling pathway, regulation of the endothelial cell apoptotic process, and negative regulation of vasculature development and morphogenesis of the epithelium were included. A total of 117 biological processes (Figure 5C) were associated with the downregulated genes, including regulation of the cell cycle, negative regulation of cell death, sprouting angiogenesis, and DNA synthesis involved in DNA repair. These data suggest an important role for TP in both the PDGF pathway and mechanisms related to angiogenesis and cell growth in PC1 cells. Thirty-one molecular functions were associated with upregulated genes (Figure 5B), and 21 were associated with downregulated genes (Figure 5D).

**Figure 6:**
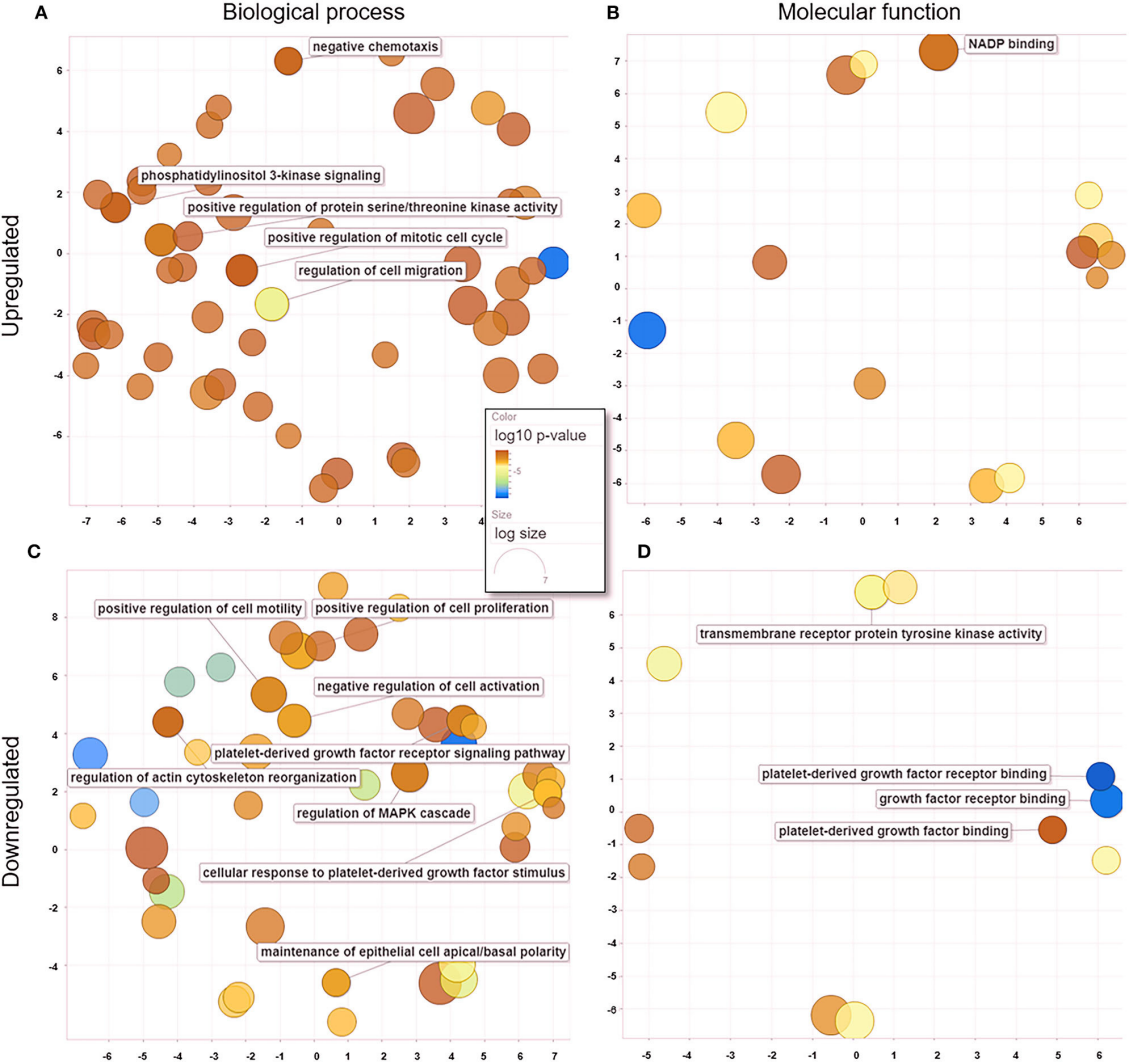
GO processes: common biological processes and molecular functions of both upregulated and downregulated DEGs in treated PC2 cells. **(A)** Biological processes of upregulated DEGs, **(B)** molecular functions of upregulated DEGs, **(C)** biological processes of downregulated DEGs, **(D)** molecular functions of downregulated DEGs. The scatter plot was produced with REVIGO software.

When comparing untreated and treated PC2 cells, we observed 18 enriched genes upregulated in treated PC2 cells, from which we identified 139 biological processes (Figure 6A) and 19 molecular functions (Figure 6B), including phosphatidylinositol 3-kinase (PI3-K) signaling, protein serine/threonine kinase activity, mitotic cell cycle regulation, and cell migration. Among the downregulated genes, 110 biological processes (Figure 6C) and 19 molecular functions (Figure 6D) were summarized and observed to be related to PDGF/PDGFR binding and signaling and regulation of cell motility and proliferation.

### PPI Network

**Figure 7:**
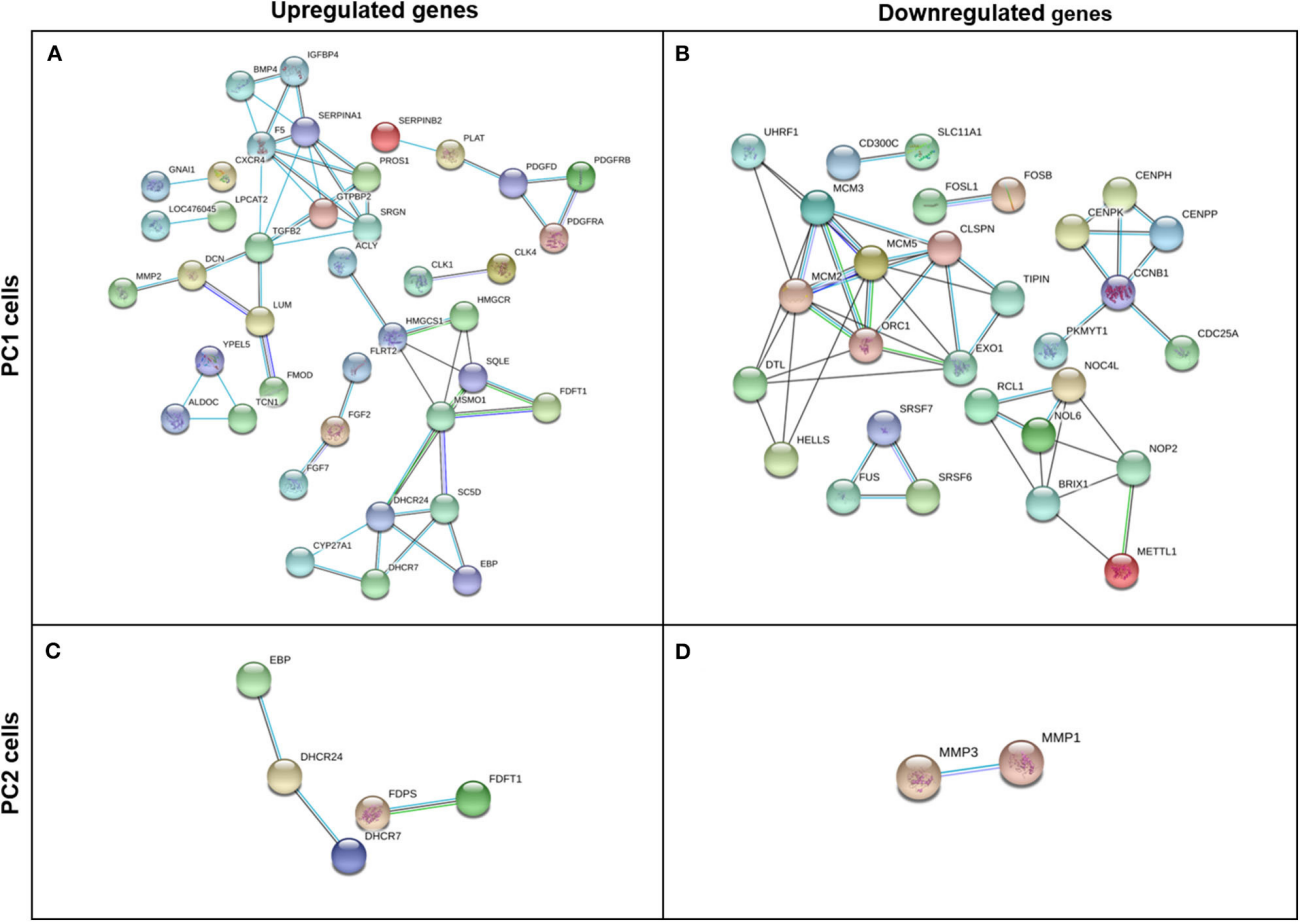
Gene interaction network of upregulated and downregulated DEGs in TP-treated PC1 (**A,B**, respectively) and PC2 cells (**C,D**, respectively) identified by STRING software using a high confidence interaction score (0.700). Circles represent genes, and lines represent protein-protein associations.

After removing any disconnected nodes in the network, PPI analysis was constructed using upregulated and downregulated genes for each cell line (PC1 and PC2). The analysis comprises a highly interactive PPI network of 170 nodes and 57 interactions in the upregulated genes in PC1 cells (Figure 7A), 77 nodes and 51 interactions in the downregulated genes in PC1 cells (Figure 7B), 18 nodes and 3 interactions in the upregulated genes in PC2 cells (Figure 7C) and 20 nodes and 1 interaction in the downregulated genes in PC2 cells (Figure 7D).

### Cell Lines Gene Expression Profile Validation in Primary Tumors

**Figure 8:**
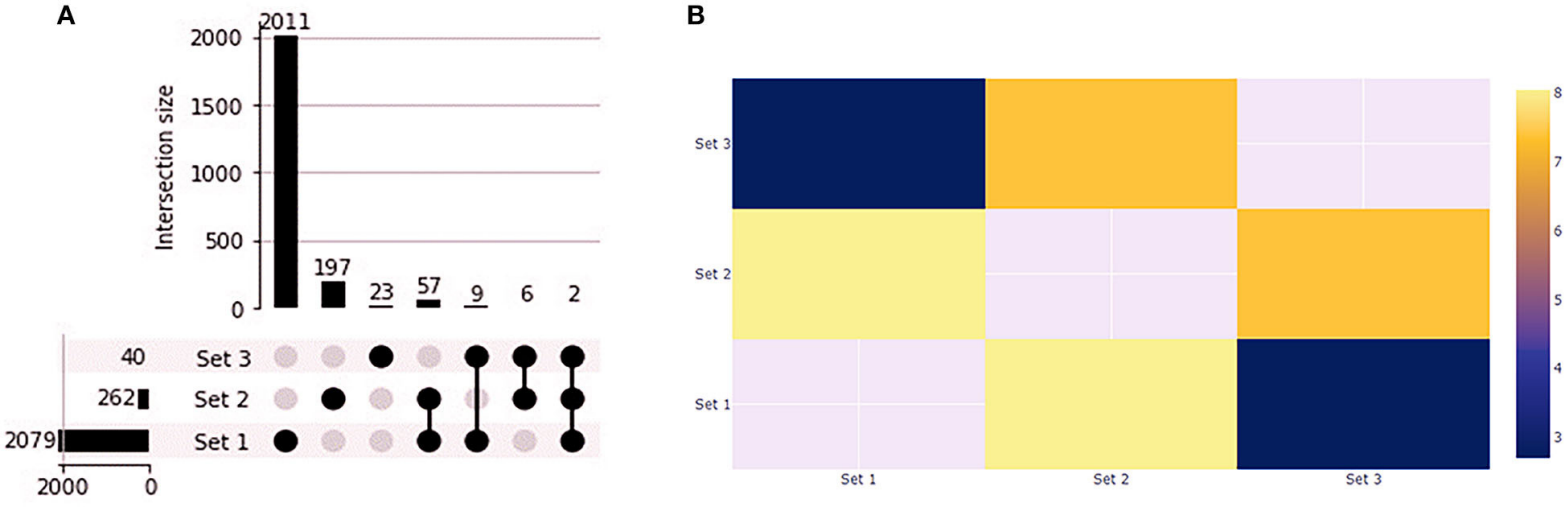
Gene expression profile of PC1 and PC2 compared to canine primary prostate tumors. **(A)** Upset plot displaying the set intersections of differentially expressed genes in the PC1 and PC2 cell lines compared to Thiemeyer et al. (23) RNA-seq dataset of canine primary prostate tumors (GSE122916). **(B)** Heatmap of Fisher's Exact test results. The –log(*p*-values) is shown in the heatmap. Each axis displays which sets are being compared and sets that cannot be compared are given a value of None. Set 1: primary tumors; Set 2: PC1; Set 3: PC2.

We found 1,412 up and 668 downregulated genes shared by the primary tumors from biopsy and needle-aspiration (23). By comparing the gene expression profile of primary tumors with the treated cell lines we observed that PC1 has 59 overlapping genes while PC2 has 11 overlapping genes (Figure 8A). Fisher exact test demonstrated the overlapping of PC1 with the primary tumors the most significant (Figure 8B). Two genes were differentially expressed in all conditions (*FABP3* and *SERPINB2*). Interestingly, 25 of 59 genes in PC1 and 6 of 11 in PC2 showed gene expression in opposite direction to that found in tumor tissues (Supplementary Table 5).

## Discussion

In the present study, we observed VEGFR2 and PDGFR-β protein expression in two primary canine PC cell lines; these receptors could represent suitable targets for therapy with RTK drugs, including TP. Deregulation of RTKs is frequently associated with different cancers and metastases (28–30). Previously, our research group investigated c-KIT expression in canine PC tissue samples and in PC1 and PC2 cells (31). In that study, we identified heterogeneous c-KIT gene and protein expression among PC tissue samples, and no KIT expression was observed in metastatic samples. In addition, we assessed KIT protein expression by western blot, and we did not find KIT expression in either PC1 or PC2 cells, confirming our IF results.

Regarding PDGFR-β and VEGFR2 expression in canine PC, we previously observed increased VEGFR2 protein expression in formalin-fixed paraffin-embedded canine PCs compared to normal prostate cells, suggesting an interesting target for TP (data not published). In humans, VEGFR2 inhibition is associated with reduced osteolysis and growth of prostate carcinoma bone metastasis (32). To the best of our knowledge, no previous study has investigated PDGFR-β in canine PC.

Although we demonstrated the absence of c-KIT protein expression in both canine PC cell lines (PC1 and PC2), we investigated *c-KIT* gene expression after TP treatment, and no significant difference was observed between the transcript levels in treated and untreated cells for either cell line. Although c-KIT protein overexpression may be correlated with more aggressive tumors, higher invasion capacity and tumor recurrence, heterogeneity and/or the absence of c-KIT protein expression has been demonstrated in the epithelial cells of human PC (31, 33–35). Moreover, the stromal microenvironment has an important role in PC biology, and increased c-KIT expression in stromal cells may affect PC development (36). In 2D cell culture, we could not verify the role of TP in the stroma; however, a previous study from our group revealed c-KIT-positive stromal cells in formalin-fixed paraffin-embedded canine PC samples (31).

PC1 and PC2 were positive for PDGFR and VEGFR receptors, but the antitumoral effect of TP was different in the cell lines. After treatment with TP, PC1 cell viability was reduced significantly (*p* < 0.05) in a dose-dependent manner, indicating drug sensitivity. In contrast, the viability of PC2 cells was not significantly altered when subjected to the same TP concentrations. These data reinforce the intertumoral heterogeneity and the importance of PC treatment planning (37). Even at higher TP concentrations (data not shown), the PC2 cell line did not show significant reductions in cell viability. In addition, the use of higher concentrations to elicit an effective response would be beyond the maximum safe dosage for *in vivo* use (38). Thus, we considered the PC2 cell line resistant to TP treatment. The global gene expression profile identified DEGs that can help to explain the resistance mechanism. We found only 17 genes in common between PC1 and PC2 cells upon comparison of the respective untreated and treated cells, indicating a difference between them after treatment with TP. Considering the differences in global gene analyses between TP-treated and untreated PC1 and PC2 cell lines, we found that *PDGF-D, PDGFR-*α, and *PDGFR-*β genes, which are involved in the PDGFR pathway, were upregulated in PC1 cells. We confirmed the upregulated expression of *PDGFR-*β in PC1 cells after treatment by qPCR. Interestingly, both cell lines presented *PDGFR-*β deregulation after TP treatment; however, the PC2 cell line showed downregulation of *PDGFR-*β. The increases in the release of RTK ligands via autocrine tumor cell production and in RTK expression are potential mechanisms of acquired resistance to tyrosine kinase inhibitors (39–41). Thus, the increased *PDGFR-*β expression in PC1 cells could be a response to the direct inhibition of this receptor as an attempt to activate the *PDGFR-*β pathway in the presence of an inhibitor (42). In addition, we observed an increase in *PDGF-D* expression, which seems to activate *PDGFR-*β without the involvement of its classical ligand, *PGDF-B*. Additionally, increased transcription of these genes has been related to tumor aggressiveness and epithelial-mesenchymal transition in multiple cancers, including PC (43–45). On the other hand, PC2 cells showed *PDGFR-*β downregulation, indicating a direct blockage of this pathway. However, associating this molecular feature with the fact that this tumor cell line is resistant to TP, PC2 cells probably did not present PDGFR-β as a driving pathway to tumor proliferation.

PC1 and PC2 cell cultures were previously established and characterized by our research group, and both cells were positive for pan-cytokeratin and prostatic specific antigen (PSA) and negative for androgen receptor (20). However, PC1 cells were derived from a nonmetastatic tumor, whereas PC2 cells were derived from a metastatic tumor (20); this expected elevation in number of gene alterations in this cell could have contributed to its resistance to TP.

In addition, we observed an increase in *VEGFR2* expression in TP-treated PC2 cells by qPCR; however, no difference was observed in the microarray analysis. An explanation for that could be the higher sensitivity of qPCR to detect small differences in expression that are undetectable by microarrays due to the flexible number of cycles in qPCR (46).

DEGs involved in the negative regulation of vasculature development and blood vessel endothelial cell proliferation (which are related to a decrease in sprouting angiogenesis) were identified in treated PC1 cells. Moreover, TP significantly affected genes involved in cell growth and activation and DNA repair by different mechanisms, for example, decreases in the mitotic cell cycle and cellular response to DNA damage stimuli. As would be expected of an inhibitor of VEGFR, PDGFR, and c-KIT kinase activity, TP can modify the expression of genes related to antiangiogenic activity and tumor growth inhibition via direct and indirect mechanisms (11, 14).

Another explanation for the resistance of PC2 cells to TP could the upregulation of genes from the PI3K pathway, which positively regulates the mitotic cell cycle, protein serine/threonine kinase activity, and cell migration. Therefore, treated PC2 cells may become resistant by activating parallel signaling pathways and mechanisms.

Dysregulated and/or elevated activation of the PI3K signaling network is one of the most common events in the oncogenesis of different cancers, including PC (47–49). In human PC, inhibition of PI3K signaling has been demonstrated to suppress invasion and induce apoptosis (50). Consistent with the main mechanism of action of TP, our results showed downregulation of genes involved in transmembrane RTK activity and PDGFR binding. The role of negative feedback in growth factor signaling pathways is to generate stability, limiting the duration and extent of signaling and preventing potentially harmful overactivation of signaling (51). However, we observed overexpression of endothelin-1 (*ET-1*), which can be a compensatory cellular mechanism that acts alone or in cooperation with other tyrosine kinase growth factors, such as PDGFR and VEGFR, to activate RTKs, leading to cell proliferation and angiogenesis (52–55). Signaling pathways require both positive and negative feedback loops to adjust their cellular response; these feedback loops act through different genes, mechanisms, and stimuli (56). Therefore, loss of homeostasis of this negative feedback can lead to oncogene activation and uncontrolled growth that can lead to cancer (57).

Migration and invasion are fundamental characteristics of cancer progression and metastasis that consequently reduce both the efficacy of therapeutics and prognosis. This study identified the overexpression of genes involved in these mechanisms, such as Rho family GTPase 1 (*RND1*) and Semaphorin 3D (*SEMA3D*). RND1 protein is concentrated at adherens junctions in both confluent fibroblasts and epithelial cells, and overexpression causes loss of cell-matrix adhesion, leading to cell rounding, which facilitates invasion (58). SEMA3D, a membrane-bound protein, is involved in cell-cell communication and plays an important role in many pathophysiological processes, such as cancer development, and its overexpression has been related to increased cell invasiveness (59, 60).

On the other hand, the expression of other genes involved in metastatic processes, such as matrix metalloproteinase 1 (*MMP1*) and 3 (*MMP3*), was downregulated. MMPs are capable of cleaving extracellular matrix protein substrates and have been identified as key factors involved in carcinogenesis and metastasis (61, 62). Among the members of the MMP family, MMP1 degrades fibrillary collagen and has been associated with invasion and poor prognosis (63). The *MMP3* gene has also been implicated as a contributor to cancer progression and reported to be responsible for inducing epithelial-mesenchymal transition and increasing cell spreading (64, 65). Our PPI network indicated an interaction between MMP1 and MMP3, which suggests a relationship between these two genes and the importance of their role in canine PC.

Interestingly, we observed downregulation of epidermal growth factor-containing fibulin-like extracellular matrix protein 1 (*EFEMP1*), which encodes a member of the fibulin family of secreted glycoproteins, and its role in carcinogenesis is controversial due to its suppressive and oncogenic activities (66). In the serum and urine of PC patients, decreased EFEMP1 expression was reported, suggesting that this protein participates in the carcinogenesis of human PC (67).

Besides, we validated our results comparing our DEGs found in treated PC cell lines with gene expression profile of primary tumors (23). Interestingly, we found gene expression in opposite direction to that found in tumor tissues and these could be interesting genes to understand the modulation of toceranib phosphate therapy in these canine prostate carcinomas. Therefore, we found *FABP3* and *SERPINB2* as common genes in our treated samples and primary prostate tumor in dogs (Supplementary Table 5), with increase of *FABP3* in all our comparative samples, but interestingly *SERPINB2* was downregulated only in PC2, which was resistant to TP. *FABP3* is related to fatty acid transport cell growth and signaling, and gene transcription (68, 69). However, its function in tumor progression still remains controversial and is described as a tumor suppressor gene in breast cancer, and associated with tumor progression in gastric carcinoma and non-small cell lung cancer (69, 70). *SERPINB2* was upregulated in treated PC1 cells as in primary canine prostate tumor, but downregulated in treated PC2 cells. *SERPINB2* overexpression has been linked with inhibition of invasion and cell migration, and prolonged survival in different cancers (71, 72). The differences between PC1 and PC2 cells can explain the differences in therapy response and *SERPINB2* could be studied in the future s as one candidate marker for TP resistance in canine prostate carcinoma.

In summary, we demonstrated that two different primary canine PC cell lines had similar patterns of RTK protein expression but had different responses to TK inhibition upon treatment with TP. Based on a global gene comparative analysis, this study revealed that TP could differentially affect genes involved in the progression of canine PC. These DEGs are important for investigating other mechanisms involved in RTK therapy and are useful targets for treating canine PC. Moreover, DEGs associated with mechanisms of cancer drug resistance were identified, including PI3K pathway, raising concerns about the development of drug resistance. Further functional studies on PI3K pathways should be carried out, that could provide useful information on possible new candidate multidrug resistance genes in canine prostate carcinoma and discovery of new drug resistance targets. The identification of the common differentially expressed genes among prostate tumor and PC1 and PC2 cells can provide further insight into the discovery of new biomarkers and genes related to resistance in canine prostate carcinoma. These findings support the biological response in some cases of canine PC and the need for more personalized cancer treatments.

## Data Availability Statement

The original Microarray dataset presented in the study are publicly available in the ArrayExpress database here: http://www.ebi.ac.uk/arrayexpress/experiments/E-MTAB-9716.

## Ethics Statement

The animal study was reviewed and approved by Ethics Committee on Animal Use (CEUA) of the School of Veterinary Medicine and Animal Science of the São Paulo State University. Written informed consent was obtained from the owners for the participation of their animals in this study.

## Author Contributions

PK executed some of the experiments, analyzed the data, prepared the figures and wrote the manuscript. PL, AL-F, and MC performed the experiments and collected data. SC performed the in silico comparative analysis from PC cell lines and data from canine PC. FD and RC analyzed and interpreted the data. CF-A conceived the project and helped with data collection and interpretation. RL-A conceived the project and participated in its design and coordination. All authors read and approved the final manuscript.

## Conflict of Interest

The authors declare that the research was conducted in the absence of any commercial or financial relationships that could be construed as a potential conflict of interest.
