# Supplementary material for: Transcriptome of Two Canine Prostate Cancer Cells Treated With Toceranib Phosphate Reveals Distinct Antitumor Profiles Associated With the PDGFR Pathway
Source: Front Vet Sci. 2020 Nov 26;7:561212. doi: 10.3389/fvets.2020.561212 (PMC7726326; doi:10.3389/fvets.2020.561212)
Supplement: Supplementary file 1 [file Table_1.DOCX]

**Supplementary table S1:** Upregulated genes in treated PC1 cells

| **Gene Symbol** | **Gene Name** | **Entrez ID** | **FC** | **p value** |
| --- | --- | --- | --- | --- |
| *INSIG1* | insulin induced gene 1 | 14300195 | 7,59 | 0,00000576 |
| *NRXN1* | neurexin 1 | 14272951 | 7,69 | 0,0000284 |
| *HMGCS1* | 3-hydroxy-3-methylglutaryl-CoA synthase 1 (soluble) | 14404882 | 4,56 | 0,0000306 |
| *PLA2G16* | phospholipase A2, group XVI | 14312826 | 3,57 | 0,0000466 |
| *TFPI2* | tissue factor pathway inhibitor 2 | 14292597 | 3,69 | 0,0000587 |
| *DCN* | decorin | 14298259 | 3,67 | 0,0000658 |
| *SLCO5A1* | solute carrier organic anion transporter family, member 5A1 | 14373210 | 3,02 | 0,000068 |
| *SLC24A2* | solute carrier family 24 (sodium/potassium/calcium exchanger), member 2 | 14277552 | 3,06 | 0,0000708 |
| *ZNF608* | zinc finger protein 608 | 14276650 | 3,04 | 0,0000738 |
| *RASL11A* | RAS-like, family 11, member A | 14355340 | 3,44 | 0,0000746 |
| *DOPEY2* | dopey family member 2 | 14384800 | 3,18 | 0,000076 |
| *LUM* | lumican | 14298255 | 8,07 | 0,0000867 |
| *FDFT1* | farnesyl-diphosphate farnesyltransferase 1 | 14355799 | 3,02 | 0,000098 |
| *LDLR* | low density lipoprotein receptor | 14335961 | 2,64 | 0,0001 |
| *TBC1D1* | TBC1 (tre-2/USP6, BUB2, cdc16) domain family, member 1 | 14379074 | 2,67 | 0,0001 |
| *EBP* | emopamil binding protein (sterol isomerase) | 14458384 | 2,68 | 0,0001 |
| *BMP4* | bone morphogenetic protein 4 | 14441454 | 2,7 | 0,0001 |
| *HBP1* | HMG-box transcription factor 1 | 14313517 | 2,77 | 0,0001 |
| *LOC490172* | interferon-induced guanylate-binding protein 1 | 14423593 | 2,77 | 0,0001 |
| *KLHL24* | kelch-like family member 24 | 14391611 | 2,84 | 0,0001 |
| *BMF* | Bcl2 modifying factor | 14381834 | 2,88 | 0,0001 |
| *TCP11L2* | t-complex 11, testis-specific-like 2 | 14272077 | 3,04 | 0,0001 |
| *AEBP1* | AE binding protein 1 | 14456550 | 3,05 | 0,0001 |
| *MOXD1* | monooxygenase, DBH-like 1 | 14261282 | 3,44 | 0,0001 |
| *NCALD* | neurocalcin delta | 14287630 | 3,7 | 0,0001 |
| *SULF1* | sulfatase 1 | 14372174 | 2,31 | 0,0002 |
| *TP53INP1* | tumor protein p53 inducible nuclear protein 1 | 14373699 | 2,33 | 0,0002 |
| *FLRT2* | fibronectin leucine rich transmembrane protein 2 | 14438980 | 2,36 | 0,0002 |
| *SERPINA1* | serpin peptidase inhibitor, clade A (alpha-1 antiproteinase, antitrypsin), member 1 | 14442716 | 2,36 | 0,0002 |
| *MMP2* | matrix metallopeptidase 2 | 14325400 | 2,49 | 0,0002 |
| *CCNG2* | cyclin G2 | 14386540 | 2,52 | 0,0002 |
| *ZC3H6* | zinc finger CCCH-type containing 6 | 14304983 | 2,53 | 0,0002 |
| *ENPP5* | ectonucleotide pyrophosphatase/phosphodiesterase 5 (putative) | 14283727 | 2,54 | 0,0002 |
| *LOC479476* | arachidonate 12-lipoxygenase, 12S-type | 14410212 | 2,54 | 0,0002 |
| *ITGA10* | integrin, alpha 10 | 14309269 | 2,56 | 0,0002 |
| *ARRDC3* | arrestin domain containing 3 | 14374105 | 2,56 | 0,0002 |
| *TGM2* | transglutaminase 2 | 14351635 | 2,57 | 0,0002 |
| *GTPBP2* | GTP binding protein 2 | 14283638 | 2,66 | 0,0002 |
| *NFIL3* | nuclear factor, interleukin 3 regulated | 14257734 | 2,76 | 0,0002 |
| *LOC491477* | carcinoembryonic antigen-related cell adhesion molecule 21 | 14259697 | 3,2 | 0,0002 |
| *LIPG* | lipase, endothelial | 14436583 | 3,74 | 0,0002 |
| *LPIN1* | lipin 1 | 14303706 | 2,18 | 0,0003 |
| *IGFBP4* | insulin-like growth factor binding protein 4 | 14451427 | 2,24 | 0,0003 |
| *PDGFR-A* | platelet-derived growth factor receptor, alpha polypeptide | 14286984 | 2,25 | 0,0003 |
| *ACSL4* | acyl-CoA synthetase long-chain family member 4 | 14463411 | 2,25 | 0,0003 |
| *LPCAT2* | lysophosphatidylcholine acyltransferase 2 | 14325383 | 2,25 | 0,0003 |
| *YPEL4* | yippee-like 4 | 14311136 | 2,26 | 0,0003 |
| *GFRA1* | GDNF family receptor alpha 1 | 14371215 | 2,3 | 0,0003 |
| *RGS2* | regulator of G-protein signaling 2 | 14401712 | 2,34 | 0,0003 |
| *TXNIP* | thioredoxin interacting protein | 14309321 | 2,38 | 0,0003 |
| *GNAI1* | guanine nucleotide binding protein (G protein), alpha inhibiting activity polypeptide 1 | 14310560 | 2,38 | 0,0003 |
| *CLK1* | CDC-like kinase 1 | 14399459 | 2,44 | 0,0003 |
| *HMGCR* | 3-hydroxy-3-methylglutaryl-CoA reductase | 14377077 | 2,48 | 0,0003 |
| *NIPAL1* | NIPA-like domain containing 1 | 14286865 | 2,58 | 0,0003 |
| *SQLE* | squalene epoxidase | 14286196 | 2,63 | 0,0003 |
| *JCHAIN* | joining chain of multimeric IgA and IgM | 14289740 | 2,65 | 0,0003 |
| *DHCR7* | 7-dehydrocholesterol reductase | 14311987 | 2,66 | 0,0003 |
| *IL1R1* | interleukin 1 receptor, type I | 14272400 | 2,67 | 0,0003 |
| *SLC6A9* | solute carrier family 6 (neurotransmitter transporter, glycine), member 9 | 14294876 | 2,74 | 0,0003 |
| *SLC24A2* | solute carrier family 24 (sodium/potassium/calcium exchanger), member 2 | 14277542 | 3,24 | 0,0003 |
| *SVIL* | supervillin | 14323596 | 2,14 | 0,0004 |
| *ACLY* | ATP citrate lyase | 14445439 | 2,15 | 0,0004 |
| *PDGFR-B* | platelet-derived growth factor receptor, beta polypeptide | 14404677 | 2,2 | 0,0004 |
| *CERCAM* | cerebral endothelial cell adhesion molecule | 14454960 | 2,21 | 0,0004 |
| *ABCC5* | ATP-binding cassette, sub-family C (CFTR/MRP), member 5 | 14392983 | 2,23 | 0,0004 |
| *PLAT* | plasminogen activator, tissue | 14300372 | 2,25 | 0,0004 |
| *PLAG1* | pleiomorphic adenoma gene 1 | 14372951 | 2,3 | 0,0004 |
| *DDIT4* | DNA-damage-inducible transcript 4 | 14403416 | 2,36 | 0,0004 |
| *TLR1* | toll-like receptor 1 | 14375983 | 2,37 | 0,0004 |
| *SUGCT* | succinyl-CoA:glutarate-CoA transferase | 14313399 | 2,41 | 0,0004 |
| *EXD1* | exonuclease 3-5 domain containing 1 | 14381939 | 2,41 | 0,0004 |
| *ARHGAP20* | Rho GTPase activating protein 20 | 14409613 | 2,69 | 0,0004 |
| *DHCR24* | 24-dehydrocholesterol reductase | 14411672 | 2,76 | 0,0004 |
| *NEIL2* | nei-like DNA glycosylase 2 | 14355811 | 2,78 | 0,0004 |
| *NR4A2* | nuclear receptor subfamily 4, group A, member 2 | 14396194 | 2,82 | 0,0004 |
| *ALDOC* | aldolase C, fructose-bisphosphate | 14453038 | 2,02 | 0,0005 |
| *NDRG2* | NDRG family member 2 | 14297825 | 2,02 | 0,0005 |
| *FGF2* | fibroblast growth factor 2 (basic) | 14318122 | 2,05 | 0,0005 |
| *SORBS1* | sorbin and SH3 domain containing 1 | 14370119 | 2,06 | 0,0005 |
| *KDM7A* | lysine (K)-specific demethylase 7A | 14299497 | 2,1 | 0,0005 |
| *C5H11orf70* | chromosome 5 open reading frame, human C11orf70 | 14415409 | 2,11 | 0,0005 |
| *CRLF1* | cytokine receptor-like factor 1 | 14329880 | 2,15 | 0,0005 |
| *CALCOCO1* | calcium binding and coiled-coil domain 1 | 14362391 | 2,31 | 0,0005 |
| *CREBRF* | CREB3 regulatory factor | 14407191 | 2,57 | 0,0005 |
| *DCLK1* | doublecortin-like kinase 1 | 14352910 | 2,58 | 0,0005 |
| *CXCR4* | chemokine (C-X-C motif) receptor 4 | 14318722 | 2,79 | 0,0005 |
| *GFRA1* | GDNF family receptor alpha 1 | 14371208 | 2,82 | 0,0005 |
| *CYP1A1* | cytochrome P450, family 1, subfamily A, polypeptide 1 | 14384142 | 3,45 | 0,0005 |
| *CCPG1* | cell cycle progression 1 | 14383106 | 2,01 | 0,0006 |
| *ADGRD1* | adhesion G protein-coupled receptor D1 | 14359850 | 2,02 | 0,0006 |
| *SREBF2* | sterol regulatory element binding transcription factor 2 | 14271290 | 2,02 | 0,0006 |
| *YPEL5* | yippee-like 5 | 14304384 | 2,02 | 0,0006 |
| *SLC6A13* | solute carrier family 6 (neurotransmitter transporter), member 13 | 14367286 | 2,05 | 0,0006 |
| *OPTN* | optineurin | 14323843 | 2,14 | 0,0006 |
| *KLHDC1* | kelch domain containing 1 | 14437504 | 2,17 | 0,0006 |
| *DKK3* | dickkopf WNT signaling pathway inhibitor 3 | 14341158 | 2,18 | 0,0006 |
| *LAMC2* | laminin, gamma 2 | 14430058 | 2,24 | 0,0006 |
| *RBL2* | retinoblastoma-like 2 | 14325432 | 2,25 | 0,0006 |
| *PRSS50* | protease, serine, 50 | 14329557 | 2,31 | 0,0006 |
| *FBXO32* | F-box protein 32 | 14288111 | 2,44 | 0,0006 |
| *PID1* | phosphotyrosine interaction domain containing 1 | 14356604 | 2,01 | 0,0007 |
| *PSD3* | pleckstrin and Sec7 domain containing 3 | 14302256 | 2,04 | 0,0007 |
| *ACSS3* | acyl-CoA synthetase short-chain family member 3 | 14295292 | 2,37 | 0,0007 |
| *FAM78B* | family with sequence similarity 78, member B | 14400913 | 2,46 | 0,0007 |
| *KLF9* | Kruppel-like factor 9 | 14257441 | 2,64 | 0,0007 |
| *SRGN* | serglycin | 14403177 | 2,65 | 0,0007 |
| *LOC479922; LOC607314* | pancreatic alpha-amylase; pancreatic alpha-amylase-like | 14456388 | 2,68 | 0,0007 |
| *KANSL1L* | KAT8 regulatory NSL complex subunit 1-like | 14399811 | 2,03 | 0,0008 |
| *FBXL2* | F-box and leucine-rich repeat protein 2 | 14346079 | 2,04 | 0,0008 |
| *NFE2L1* | nuclear factor, erythroid 2-like 1 | 14446155 | 2,05 | 0,0008 |
| *GSAP* | gamma-secretase activating protein | 14313677 | 2,08 | 0,0008 |
| *CHAC1* | ChaC glutathione-specific gamma-glutamylcyclotransferase 1 | 14379835 | 2,12 | 0,0008 |
| *FAXDC2* | fatty acid hydroxylase domain containing 2 | 14404448 | 2,39 | 0,0008 |
| *TGFB2* | transforming growth factor, beta 2 | 14400771 | 2,52 | 0,0008 |
| *SC5D* | sterol-C5-desaturase | 14414360 | 2,54 | 0,0008 |
| *ACP5* | acid phosphatase 5, tartrate resistant | 14330808 | 2,54 | 0,0008 |
| *MIR29C-1; MIR29C-2* | microRNA mir-29c-1; microRNA mir-29c-2 | 14433548 | 2,55 | 0,0008 |
| *MIR29C-1; MIR29C-2* | microRNA mir-29c-1; microRNA mir-29c-2 | 14433550 | 2,55 | 0,0008 |
| *SYTL2* | synaptotagmin-like 2 | 14337697 | 2,73 | 0,0008 |
| *PDCD4* | programmed cell death 4 (neoplastic transformation inhibitor) | 14368790 | 2,03 | 0,0009 |
| *DNAJB4* | DnaJ (Hsp40) homolog, subfamily B, member 4 | 14428812 | 2,09 | 0,0009 |
| *MIR8810; AMPD3* | microRNA mir-8810; adenosine monophosphate deaminase 3 | 14338927 | 2,12 | 0,0009 |
| *BCL6* | B-cell CLL/lymphoma 6 | 14393213 | 2,18 | 0,0009 |
| *JMY* | junction mediating and regulatory protein, p53 cofactor | 14376901 | 2,23 | 0,0009 |
| *STK38L* | serine/threonine kinase 38 like | 14366257 | 2,26 | 0,0009 |
| *TCN1* | transcobalamin I (vitamin B12 binding protein, R binder family) | 14341603 | 2,29 | 0,0009 |
| *MUC15* | mucin 15, cell surface associated | 14341505 | 2,06 | 0,0011 |
| *FUT1* | fucosyltransferase 1 (galactoside 2-alpha-L-fucosyltransferase, H blood group) | 14259031 | 2,08 | 0,0011 |
| *SAT1* | spermidine/spermine N1-acetyltransferase 1 | 14457690 | 2,59 | 0,0011 |
| *EDNRA* | endothelin receptor type A | 14296014 | 2,74 | 0,0011 |
| *MAML3* | mastermind-like transcriptional coactivator 3 | 14316870 | 2,04 | 0,0012 |
| *ZNF395* | zinc finger protein 395 | 14353660 | 2,11 | 0,0012 |
| *SELL* | selectin L | 14430659 | 2,99 | 0,0012 |
| *CLK4* | CDC-like kinase 4 | 14273866 | 2,24 | 0,0013 |
| *CERS3* | ceramide synthase 3 | 14374679 | 2,42 | 0,0014 |
| *ERAP2* | endoplasmic reticulum aminopeptidase 2 | 14376482 | 2,89 | 0,0014 |
| *FABP3* | fatty acid binding protein 3, muscle and heart | 14321832 | 2,05 | 0,0015 |
| *SMOC2* | SPARC related modular calcium binding 2 | 14256513 | 2,06 | 0,0015 |
| *SGK494* | uncharacterized serine/threonine-protein kinase SgK494 | 14453076 | 2,01 | 0,0017 |
| *SLC16A2* | solute carrier family 16, member 2 (thyroid hormone transporter) | 14459088 | 2,21 | 0,0017 |
| *LOC612019; LOC479922; LOC480825; LOC607314* | pancreatic alpha-amylase; pancreatic alpha-amylase-like | 14455986 | 2,35 | 0,0017 |
| *PDGF-D* | platelet derived growth factor D | 14409780 | 2,47 | 0,0017 |
| *DLA-DRA* | MHC class II DR alpha chain | 14279695 | 2,93 | 0,0018 |
| *PCDH7* | protocadherin 7 | 14379107 | 2,09 | 0,0019 |
| *FGF7* | fibroblast growth factor 7 | 14380425 | 2,68 | 0,0019 |
| *KLHL15* | kelch-like family member 15 | 14461586 | 2,18 | 0,002 |
| *FMOD* | fibromodulin | 14401381 | 2,03 | 0,0021 |
| *FAT4* | FAT atypical cadherin 4 | 14318073 | 2,04 | 0,0022 |
| *LOC607276; LOC612019* | pancreatic alpha-amylase-like; pancreatic alpha-amylase | 14455969 | 2,44 | 0,0022 |
| *DHRS2* | dehydrogenase/reductase (SDR family) member 2 | 14436903 | 2,07 | 0,0025 |
| *F5* | coagulation factor V (proaccelerin, labile factor) | 14430684 | 2,09 | 0,0025 |
| *SUGCT* | succinyl-CoA:glutarate-CoA transferase | 14313394 | 2,04 | 0,0026 |
| *SERPINF1* | serpin peptidase inhibitor, clade F (alpha-2 antiplasmin, pigment epithelium derived factor), member 1 | 14447713 | 2,71 | 0,0026 |
| *RSRP1* | arginine/serine-rich protein 1 | 14322184 | 2,14 | 0,0027 |
| *MCOLN3* | mucolipin 3 | 14423752 | 2,33 | 0,0027 |
| *ELOVL7* | ELOVL fatty acid elongase 7 | 14324780 | 2,3 | 0,003 |
| *LOC100685565* | tetratricopeptide repeat protein 39B-like | 14304668 | 2,35 | 0,003 |
| *LOC607460; LOC612019* | pancreatic alpha-amylase | 14456239 | 2,55 | 0,003 |
| *LOC102154725* | methylsterol monooxygenase 1 pseudogene | 14457299 | 3,4 | 0,0031 |
| *FAM35A* | family with sequence similarity 35, member A | 14403840 | 2,06 | 0,0032 |
| *CHL1* | cell adhesion molecule L1-like | 14333480 | 2,49 | 0,0034 |
| *TNFAIP3* | tumor necrosis factor, alpha-induced protein 3 | 14255559 | 2,03 | 0,0035 |
| *TESK2* | testis-specific kinase 2 | 14294737 | 2,1 | 0,0036 |
| *SERPINB2* | serpin peptidase inhibitor, clade B (ovalbumin), member 2 | 14260883 | 2,27 | 0,0037 |
| *PDE3A* | phosphodiesterase 3A, cGMP-inhibited | 14366416 | 2,35 | 0,0041 |
| *CYP27A1* | cytochrome P450, family 27, subfamily A, polypeptide 1 | 14398789 | 2,35 | 0,005 |
| *CALCRL* | calcitonin receptor-like | 14397517 | 2,12 | 0,0051 |
| *LRCH2* | leucine-rich repeats and calponin homology (CH) domain containing 2 | 14463516 | 2,04 | 0,0052 |
| *VLDLR* | very low density lipoprotein receptor | 14257570 | 2,02 | 0,0055 |
| *LOC100855970* | protocadherin beta-4 | 14320227 | 2,01 | 0,006 |
| *RARRES3* | retinoic acid receptor responder (tazarotene induced) 3 | 14316455 | 2,28 | 0,0063 |
| *CFAP69* | cilia and flagella associated protein 69 | 14290438 | 2,03 | 0,0067 |
| *MIR125B-1* | microRNA mir-125b-1 | 14409053 | 2,48 | 0,0072 |
| *KLHL28* | kelch-like family member 28 | 14441087 | 2,28 | 0,0075 |
| *MTURN* | maturin, neural progenitor differentiation regulator homolog (Xenopus) | 14291266 | 2,12 | 0,0078 |
| *PROS1* | protein S (alpha) | 14389903 | 2,31 | 0,0082 |
| *GLRB* | glycine receptor, beta | 14296277 | 2,45 | 0,0084 |
| *MPPED2* | metallophosphoesterase domain containing 2 | 14310985 | 2,02 | 0,0101 |
| *CDS1* | CDP-diacylglycerol synthase (phosphatidate cytidylyltransferase) 1 | 14386750 | 2,05 | 0,0152 |
| *OLR1* | oxidized low density lipoprotein (lectin-like) receptor 1 | 14364302 | 2,03 | 0,0157 |
| *MEST* | mesoderm specific transcript | 14292024 | 2,36 | 0,0161 |
| *OR10A12* | olfactory receptor | 14330388 | 2,13 | 0,0166 |
| *DHRS1* | dehydrogenase/reductase (SDR family) member 1 | 14440617 | 2,05 | 0,0169 |
| *LOC100682624* | olfactory receptor 4C11-like | 14456199 | 2,12 | 0,042 |
