# Supplementary material for: Transcriptome of Two Canine Prostate Cancer Cells Treated With Toceranib Phosphate Reveals Distinct Antitumor Profiles Associated With the PDGFR Pathway
Source: Front Vet Sci. 2020 Nov 26;7:561212. doi: 10.3389/fvets.2020.561212 (PMC7726326; doi:10.3389/fvets.2020.561212)
Supplement: Supplementary file 2 [file Table_2.DOCX]

**Supplementary table S2:** Downregulated genes in treated PC1 cells

| **Gene Symbol** | **Gene Name** | **Entrez ID** | **FC** | **p value** |
| --- | --- | --- | --- | --- |
| *LOC612564* | membrane-spanning 4-domains subfamily A member 7 | 14339519 | -3,55 | 5,05E-05 |
| *SRSF6* | serine/arginine-rich splicing factor 6 | 14349213 | -3,2 | 8,88E-05 |
| *ID1* | inhibitor of DNA binding 1, dominant negative helix-loop-helix protein | 14348293 | -2,79 | 0,0001 |
| *CCNB1* | cyclin B1 | 14320839 | -2,54 | 0,0001 |
| *ABCA1* | ATP-binding cassette, sub-family A (ABC1), member 1 | 14278443 | -3,31 | 0,0002 |
| *CLDN10* | claudin 10 | 14342285 | -2,6 | 0,0002 |
| *SPINK5* | serine peptidase inhibitor, Kazal type 5 | 14320425 | -3,17 | 0,0003 |
| *LOC606761* | cell division cycle-associated protein 7 | 14354874 | -2,51 | 0,0003 |
| *RANBP1* | RAN binding protein 1 | 14361940 | -2,18 | 0,0003 |
| *CDCA7* | cell division cycle associated 7 | 14395825 | -3,26 | 0,0004 |
| *LOC485710* | uncharacterized LOC485710 | 14347297 | -3,17 | 0,0004 |
| *VGLL2* | vestigial-like family member 2 | 14256601 | -2,86 | 0,0004 |
| *SBSPON* | somatomedin B and thrombospondin, type 1 domain containing | 14373323 | -2,68 | 0,0004 |
| *CD3EAP* | CD3e molecule, epsilon associated protein | 14264838 | -2,3 | 0,0004 |
| *FOSL1* | FOS-like antigen 1 | 14312391 | -2,29 | 0,0004 |
| *FUS* | FUS RNA binding protein | 14425550 | -2,26 | 0,0004 |
| *METTL1* | methyltransferase like 1 | 14270434 | -2,23 | 0,0004 |
| *RNASEH2B* | ribonuclease H2, subunit B | 14342861 | -2,11 | 0,0004 |
| *E2F1* | E2F transcription factor 1 | 14351236 | -2,07 | 0,0004 |
| *GINS3* | GINS complex subunit 3 (Psf3 homolog) | 14325046 | -2,48 | 0,0005 |
| *LOC100688091* | uncharacterized LOC100688091 | 14312880 | -2,19 | 0,0005 |
| *MRTO4* | MRT4 homolog, ribosome maturation factor | 14326635 | -2,1 | 0,0005 |
| *SASS6* | SAS-6 centriolar assembly protein | 14423255 | -2,06 | 0,0005 |
| *ANKRD1* | ankyrin repeat domain 1 (cardiac muscle) | 14369962 | -4,16 | 0,0006 |
| *LYVE1* | lymphatic vessel endothelial hyaluronan receptor 1 | 14341081 | -2,21 | 0,0006 |
| *DTD2* | D-tyrosyl-tRNA deacylase 2 (putative) | 14440870 | -2,15 | 0,0007 |
| *TOMM40* | translocase of outer mitochondrial membrane 40 homolog (yeast) | 14264954 | -2,1 | 0,0007 |
| *NAF1* | nuclear assembly factor 1 ribonucleoprotein | 14299058 | -2,02 | 0,0007 |
| *TIPIN* | TIMELESS interacting protein | 14383783 | -2,02 | 0,0007 |
| *TIGAR* | TP53 induced glycolysis regulatory phosphatase | 14367167 | -2,23 | 0,0008 |
| *E2F8* | E2F transcription factor 8 | 14341467 | -2,4 | 0,0009 |
| *LOC609800* | CMRF35-like molecule 6 | 14444226 | -2,01 | 0,0009 |
| *CENPP* | centromere protein P | 14263476 | -2,2 | 0,001 |
| *UNG* | uracil DNA glycosylase | 14361237 | -3,12 | 0,0011 |
| *FAM83A* | family with sequence similarity 83, member A | 14286103 | -2,02 | 0,0011 |
| *QTRTD1* | queuine tRNA-ribosyltransferase domain containing 1 | 14389366 | -2,2 | 0,0013 |
| *SRSF7* | serine/arginine-rich splicing factor 7 | 14307855 | -2,13 | 0,0013 |
| *POLR3F* | polymerase (RNA) III (DNA directed) polypeptide F, 39 kDa | 14350460 | -2,03 | 0,0014 |
| *NPY* | neuropeptide Y | 14291140 | -2,13 | 0,0015 |
| *TMEM201* | transmembrane protein 201 | 14412387 | -2,23 | 0,0016 |
| *CABYR* | calcium binding tyrosine-(Y)-phosphorylation regulated | 14435923 | -2,16 | 0,0016 |
| *CDC25A* | cell division cycle 25A | 14329359 | -2,38 | 0,0017 |
| *ESM1* | endothelial cell-specific molecule 1 | 14404773 | -2,78 | 0,0019 |
| *SLC11A1* | solute carrier family 11 (proton-coupled divalent metal ion transporter), member 1 | 14398682 | -2,71 | 0,0019 |
| *LOC484356* | uncharacterized LOC484356 | 14258668 | -2,22 | 0,0021 |
| *CENPK* | centromere protein K | 14324857 | -2,4 | 0,0022 |
| *GEM* | GTP binding protein overexpressed in skeletal muscle | 14373638 | -2,13 | 0,0022 |
| *HMOX1* | heme oxygenase 1 | 14271904 | -2,62 | 0,0023 |
| *AMD1* | adenosylmethionine decarboxylase 1 | 14282088 | -2,03 | 0,0023 |
| *PKMYT1* | protein kinase, membrane associated tyrosine/threonine 1 | 14422314 | -2,18 | 0,0025 |
| *DTL* | denticleless E3 ubiquitin protein ligase homolog (Drosophila) | 14429668 | -2,01 | 0,0026 |
| *FOSB* | FBJ murine osteosarcoma viral oncogene homolog B | 14264833 | -2,47 | 0,0027 |
| *WNT2B* | wingless-type MMTV integration site family, member 2B | 14309822 | -2,24 | 0,0029 |
| *CEP55* | centrosomal protein 55kDa | 14367893 | -2,17 | 0,0033 |
| *CENPH* | centromere protein H | 14320843 | -2,36 | 0,004 |
| *SPP1* | secreted phosphoprotein 1 | 14386892 | -2,24 | 0,004 |
| *OGN* | osteoglycin | 14257864 | -2,09 | 0,004 |
| *NOC4L* | nucleolar complex associated 4 homolog | 14359746 | -2,7 | 0,0043 |
| *EXO1* | exonuclease 1 | 14430897 | -2,33 | 0,0043 |
| *NOP2* | NOP2 nucleolar protein | 14364687 | -2,23 | 0,0044 |
| *UHRF1* | ubiquitin-like with PHD and ring finger domains 1 | 14336719 | -2,55 | 0,0045 |
| *NOL6* | nucleolar protein 6 (RNA-associated) | 14277793 | -2,12 | 0,0052 |
| *ORC1* | origin recognition complex, subunit 1 | 14294361 | -2,48 | 0,006 |
| *HNRNPDL* | heterogeneous nuclear ribonucleoprotein D-like | 14387831 | -2,41 | 0,0064 |
| *RCL1* | RNA terminal phosphate cyclase-like 1 | 14257624 | -2,24 | 0,0065 |
| *PSMC3IP; FAM134C* | PSMC3 interacting protein; family with sequence similarity 134, member C | 14445295 | -2,09 | 0,0065 |
| *SKA3* | spindle and kinetochore associated complex subunit 3 | 14353285 | -2,05 | 0,0068 |
| *WFDC5* | WAP four-disulfide core domain 5 | 14351820 | -2,02 | 0,0068 |
| *BRIX1* | BRX1, biogenesis of ribosomes | 14408426 | -2,19 | 0,0072 |
| *MCM2* | minichromosome maintenance complex component 2 | 14327242 | -2,6 | 0,0073 |
| *MIR98* | microRNA mir-98 | 14462430 | -2,44 | 0,0073 |
| *LOC486670* | histone H4 | 14364118 | -2,12 | 0,0079 |
| *MCM5* | minichromosome maintenance complex component 5 | 14271891 | -2,87 | 0,0082 |
| *LOC486484* | olfactory receptor 6C75-like | 14365165 | -2,02 | 0,0084 |
| *CLSPN* | claspin | 14294119 | -2,19 | 0,0102 |
| *SLC25A19* | solute carrier family 25 (mitochondrial thiamine pyrophosphate carrier), member 19 | 14444093 | -2,09 | 0,0102 |
| *PIGW* | phosphatidylinositol glycan anchor biosynthesis, class W | 14446944 | -2,02 | 0,0134 |
| *HELLS* | helicase, lymphoid-specific | 14367993 | -2,17 | 0,0147 |
| *TRIP13* | thyroid hormone receptor interactor 13 | 14392722 | -2,08 | 0,0166 |
| *MCM3* | minichromosome maintenance complex component 3 | 14283972 | -2,09 | 0,0184 |
| *HSPE1* | heat shock 10kDa protein 1 (chaperonin 10) | 14397790 | -2,02 | 0,0205 |
| *AIMP1* | aminoacyl tRNA synthetase complex-interacting multifunctional protein 1 | 14387277 | -2,08 | 0,0279 |
