# Supplementary material for: Transcriptome of Two Canine Prostate Cancer Cells Treated With Toceranib Phosphate Reveals Distinct Antitumor Profiles Associated With the PDGFR Pathway
Source: Front Vet Sci. 2020 Nov 26;7:561212. doi: 10.3389/fvets.2020.561212 (PMC7726326; doi:10.3389/fvets.2020.561212)
Supplement: Supplementary file 3 [file Table_3.DOCX]

**Supplementary table S3:** Upregulated genes in treated PC2 cells

| **Gene Symbol** | **Gene Name** | **Entrez ID** | **FC** | **p value** |
| --- | --- | --- | --- | --- |
| *DHCR24* | 24-dehydrocholesterol reductase | 14411672 | 3,2 | 8,45E-05 |
| *NEXN* | nexilin (F actin binding protein) | 14428818 | 2,84 | 0,0002 |
| *FDFT1* | farnesyl-diphosphate farnesyltransferase 1 | 14355799 | 2,58 | 0,0002 |
| *EDN1* | endothelin 1 | 14393998 | 2,54 | 0,0002 |
| *DHCR7* | 7-dehydrocholesterol reductase | 14311987 | 2,19 | 0,0002 |
| *FABP3* | fatty acid binding protein 3, muscle and heart | 14321832 | 2,41 | 0,0003 |
| *FDPS* | farnesyl diphosphate synthase | 14435210 | 2,17 | 0,0004 |
| *EBP* | emopamil binding protein (sterol isomerase) | 14458384 | 2,03 | 0,0005 |
| *PRTFDC1* | phosphoribosyl transferase domain containing 1 | 14319232 | 2,13 | 0,0008 |
| *OCLN* | occludin | 14320896 | 2,01 | 0,001 |
| *OMD* | osteomodulin | 14257861 | 2,3 | 0,0012 |
| *OLFM3* | olfactomedin 3 | 14423212 | 2,13 | 0,0015 |
| *SEMA3D* | sema domain, immunoglobulin domain (Ig), short basic domain, secreted, (semaphorin) 3D | 14313894 | 2,32 | 0,0017 |
| *CEMIP* | cell migration inducing protein, hyaluronan binding | 14378163 | 2,11 | 0,002 |
| *LOC612207* | insulin growth factor-like family member 3 | 14259320 | 2,02 | 0,002 |
| *IDH1* | isocitrate dehydrogenase 1 (NADP+), soluble | 14399798 | 2,19 | 0,0044 |
| *RND1* | Rho family GTPase 1 | 14363003 | 2,04 | 0,0048 |
| *CCL17* | chemokine (C-C motif) ligand 17 | 14325178 | 2,21 | 0,005 |
