# Supplementary material for: Transcriptome of Two Canine Prostate Cancer Cells Treated With Toceranib Phosphate Reveals Distinct Antitumor Profiles Associated With the PDGFR Pathway
Source: Front Vet Sci. 2020 Nov 26;7:561212. doi: 10.3389/fvets.2020.561212 (PMC7726326; doi:10.3389/fvets.2020.561212)
Supplement: Supplementary file 4 [file Table_4.DOCX]

**Supplementary table S4:** Downregulated genes in treated PC2 cells

| **Gene Symbol** | **Gene Name** | **Entrez ID** | **FC** | **p value** |
| --- | --- | --- | --- | --- |
| *HAS2* | hyaluronan synthase 2 | 14288059 | -3,12 | 3,27E-05 |
| *TMEM26* | transmembrane protein 26 | 14406114 | -3,12 | 5,69E-05 |
| *CBLN4* | cerebellin 4 precursor | 14352298 | -3,02 | 0,000066 |
| *EFEMP1* | EGF containing fibulin-like extracellular matrix protein 1 | 14273152 | -2,63 | 7,38E-05 |
| *LAMA4* | laminin, alpha 4 | 14285468 | -2,73 | 8,32E-05 |
| *TRIB2* | tribbles pseudokinase 2 | 14303727 | -2,71 | 8,54E-05 |
| *MMP3* | matrix metallopeptidase 3 (stromelysin 1, progelatinase) | 14409820 | -3,04 | 0,0001 |
| *SMIM3* | small integral membrane protein 3 | 14407764 | -2,69 | 0,0001 |
| *IL7R* | interleukin 7 receptor | 14408372 | -2,57 | 0,0001 |
| *IL1R1* | interleukin 1 receptor, type I | 14272400 | -2,77 | 0,0002 |
| *SEMA6D* | sema domain, transmembrane domain (TM), and cytoplasmic domain, (semaphorin) 6D | 14380344 | -2,27 | 0,0002 |
| *PDGFR-A* | platelet-derived growth factor receptor, alpha polypeptide | 14286984 | -2,24 | 0,0002 |
| *MMP1* | matrix metallopeptidase 1 | 14409830 | -3,57 | 0,0003 |
| *SERPINB2* | serpin peptidase inhibitor, clade B (ovalbumin), member 2 | 14260883 | -2,49 | 0,0003 |
| *IL18RAP* | interleukin 18 receptor accessory protein | 14272354 | -2,15 | 0,0003 |
| *SLPI* | secretory leukocyte peptidase inhibitor | 14351829 | -2,01 | 0,0008 |
| *MIR33B* | microRNA mir-33b | 14411283 | -2,19 | 0,0011 |
| *SLC6A2* | solute carrier family 6 (neurotransmitter transporter), member 2 | 14325370 | -2,09 | 0,0014 |
| *LIN7A* | lin-7 homolog A (C. elegans) | 14298069 | -2,07 | 0,0014 |
| *ACTC1* | actin, alpha, cardiac muscle 1 | 14381724 | -2,49 | 0,0025 |
| *CLIC6* | chloride intracellular channel 6 | 14384771 | -2,11 | 0,0059 |
| *LOC607368* | Ig lambda chain V-I region BL2 | 14358995 | -2,26 | 0,0087 |
