# Supplementary material for: Transcriptome of Two Canine Prostate Cancer Cells Treated With Toceranib Phosphate Reveals Distinct Antitumor Profiles Associated With the PDGFR Pathway
Source: Front Vet Sci. 2020 Nov 26;7:561212. doi: 10.3389/fvets.2020.561212 (PMC7726326; doi:10.3389/fvets.2020.561212)
Supplement: Supplementary file 5 [file Table_5.DOCX]

**Supplementary table S5:** Log Fold-Change values of differentially expressed genes (DEG) in canine prostate samples (biopsy and needle aspiration samples), treated PC1 and PC2 cells.

| **DEG** | **Canine PC (biopsy)** | **Canine PC (needle)** | **PC1** | **PC2** |
| --- | --- | --- | --- | --- |
| FABP3 | 6,7181 | 5,2035 | 1,035624 | 1,269033 |
| SERPINB2 | 4,6005 | 4,351 | 1,182692 | -1,31615 |
| SPINK5 | -8,8504 | -7,4669 | -1,66448 |  |
| CLDN10 | -7,138 | -14,422 | -1,37851 |  |
| PRSS50 | -6,787 | -5,1405 | 1,207893 |  |
| OGN | -3,6502 | -6,2864 | -1,0635 |  |
| PDE3A | -2,5109 | -5,8846 | 1,232661 |  |
| CDS1 | -2,3077 | -2,5493 | 1,035624 |  |
| FAM78B | -2,1968 | -6,2474 | 1,298658 |  |
| RSRP1 | -1,72 | -1,5181 | 1,097611 |  |
| GTPBP2 | -1,5779 | -2,238 | 1,411426 |  |
| DHRS1 | -1,5536 | -1,3146 | 1,035624 |  |
| SRGN | 8,2041 | 4,9947 | 1,405992 |  |
| FAM83A | 8,1255 | 10,112 | -1,01436 |  |
| DHRS2 | 7,4984 | 7,2803 | 1,049631 |  |
| SYTL2 | 7,3214 | 5,6329 | 1,448901 |  |
| CERS3 | 6,9739 | 6,0425 | 1,275007 |  |
| OLR1 | 6,7813 | 5,3153 | 1,02148 |  |
| F5 | 6,7396 | 4,2773 | 1,063503 |  |
| CEP55 | 6,4559 | 4,5939 | -1,1177 |  |
| LDLR | 6,0424 | 4,1241 | 1,400538 |  |
| SELL | 5,8059 | 4,0275 | 1,580145 |  |
| FOSB | 5,78 | 3,406 | -1,30451 |  |
| FOSL1 | 5,7787 | 4,6194 | -1,19535 |  |
| LOC609800 | 5,6644 | 5,1914 | -1,0072 |  |
| LAMC2 | 5,613 | 8,6588 | 1,163499 |  |
| LIPG | 5,5126 | 5,5218 | 1,903038 |  |
| SLCO5A1 | 5,4069 | 3,7324 | 1,594549 |  |
| CDCA7 | 4,9489 | 4,2377 | -1,70487 |  |
| ERAP2 | 4,9335 | 3,6992 | 1,531069 |  |
| DLA-DRA | 4,7697 | 3,6428 | 1,550901 |  |
| DTL | 4,6072 | 3,3737 | -1,0072 |  |
| TGM2 | 4,5866 | 9,1505 | 1,361768 |  |
| SERPINF1 | 4,5865 | 3,2515 | 1,438293 |  |
| FLRT2 | 4,4322 | 8,1854 | 1,238787 |  |
| TLR1 | 3,8286 | 4,7057 | 1,244887 |  |
| CENPH | 3,7305 | 2,3553 | -1,23879 |  |
| MCM5 | 3,6935 | 3,3157 | -1,52105 |  |
| CCNB1 | 3,4774 | 3,0251 | -1,34483 |  |
| LUM | 3,4344 | 4,2983 | 3,012569 |  |
| MMP2 | 3,2914 | 2,5737 | 1,316146 |  |
| NIPAL1 | 3,2704 | 2,3487 | 1,367371 |  |
| UHRF1 | 3,2432 | 2,3345 | 1,350497 |  |
| CXCR4 | 3,2326 | 3,3449 | 1,480265 |  |
| SQLE | 3,232 | 3,2786 | 1,395063 |  |
| UNG | 2,9943 | 2,7131 | -1,64155 |  |
| VLDLR | 2,9451 | 4,6899 | 1,014355 |  |
| PLAT | 2,798 | 5,6213 | 1,169925 |  |
| MCM3 | 2,7131 | 1,8728 | -1,0635 |  |
| IGFBP4 | 2,6709 | 3,9319 | 1,163499 |  |
| STK38L | 2,5291 | 2,9006 | 1,176323 |  |
| HELLS | 2,4614 | 1,6222 | -1,1177 |  |
| HMGCS1 | 2,3705 | 1,6701 | 2,189034 |  |
| SLC11A1 | 2,2836 | 2,9882 | -1,43829 |  |
| TIGAR | 1,9707 | 1,699 | -1,15704 |  |
| ABCA1 | 1,8419 | 1,9784 | -1,72683 |  |
| PKMYT1 | 1,8287 | 2,3134 | -1,12433 |  |
| RGS2 | 1,6984 | 3,9402 | 1,226509 |  |
| RANBP1 | 1,2833 | 1,4614 | -1,12433 |  |
| CLIC6 | -3,445 | -8,534 |  | -1,07724 |
| OMD | -2,8726 | -7,6949 |  | 1,201634 |
| MMP1 | 12,334 | 9,4521 |  | -1,83592 |
| CCL17 | 9,2269 | 5,5317 |  | 1,144046 |
| HAS2 | 7,0455 | 7,4057 |  | -1,64155 |
| LOC612207 | 6,3886 | 5,7735 |  | 1,014355 |
| IL7R | 3,6412 | 2,9507 |  | -1,36177 |
| IL18RAP | 3,4717 | 5,3476 |  | -1,10434 |
| IDH1 | 2,8344 | 1,8297 |  | 1,130931 |
